# A cytokinetic ring-driven cell rotation achieves Hertwig’s rule in early development

**DOI:** 10.1073/pnas.2318838121

**Published:** 2024-06-13

**Authors:** Teije C. Middelkoop, Jonas Neipel, Caitlin E. Cornell, Ronald Naumann, Lokesh G. Pimpale, Frank Jülicher, Stephan W. Grill

**Affiliations:** ^a^Max Planck Institute of Molecular Cell Biology and Genetics, 01307 Dresden, Germany; ^b^Laboratory of Developmental Mechanobiology, Division Biocev, Institute of Molecular Genetics of the Czech Academy of Sciences, 14220 Prague, Czech Republic; ^c^Max Planck Institute for the Physics of Complex Systems, 01187 Dresden, Germany; ^d^Department of Bioengineering, University of California, Berkeley, CA 94720; ^e^Cluster of Excellence Physics of Life, Technical University Dresden, 01062 Dresden, Germany

**Keywords:** cytokinesis, actomyosin, biophysics, cell biology, development

## Abstract

Hertwig’s rule states that cells divide along their longest axis, usually driven by forces acting on the mitotic spindle. Here, we show that in contrast to this rule, microtubule-based pulling forces in early *Caenorhabditis elegans* embryos align the spindle with the short axis of the cell. We combine theory with experiments to reveal that in order to correct this misalignment, inward forces generated by the constricting cytokinetic ring rotate the entire cell until the spindle is aligned with the cell’s long axis. Experiments with slightly compressed mouse zygotes indicate that this cytokinetic ring-driven mechanism of ensuring Hertwig’s rule is general for cells capable of rotating inside a confining shell, a scenario that applies to early cell divisions of many systems.

## Significance

Animal cells have a general tendency to divide along their geometric long axis, a phenomenon discovered by embryologist Oscar Hertwig in the late 19th century. We here report a physical mechanism by which Hertwig’s rule is executed in early embryos. By combining physical theory with experiments in *Caenorhabditis elegans* and mouse embryos, we show that myosin-dependent forces that drive the ingression of the cytokinetic ring also drive a whole cell rotation. This rotation stops when the cell division axis is aligned with the geometric long axis, thereby executing Hertwig’s rule.

Many cells divide along their long axis, as first described by Oscar Hertwig in 1884 (1, 2). This discovery builds on earlier work from Sachs concerning orientations of subsequent cell divisions (3, 4) and is since known as Hertwig’s long axis rule (5–12). Since the mitotic spindle is bisected during cell division for proper chromosome segregation, the axis along which the mitotic spindle is oriented determines the axis of cell division (13). Hence, the orientation of the mitotic spindle determines the axis along which a cell is divided. But how then does the mitotic spindle align with the long axis of a cell, thereby facilitating Hertwig’s rule?

If misaligned, moving the spindle to orient it with the long axis of the cell requires force generation. What types of forces can act upon microtubules and microtubule asters? We distinguish force generation inside the cell, within the cytoplasm, from force generation at the cell cortex. First, within the cytoplasm, dynein motors that are attached to cytoplasmic anchors can exert forces on microtubules (14–17). In the case that anchors are present evenly throughout the cytoplasm, the net force generated onto an astral microtubule is proportional to its length. It is clear that microtubule asters can position themselves at the center of a cell through this mechanism of length-dependent force generation onto microtubules in the cytoplasm (5, 14, 16, 18–21).

Second, microtubules that grow toward the periphery can continue to do so as they encounter the cell surface, which gives rise to a pushing force (22, 23). Collectively, pushing by astral microtubules can give rise to an elastic restoring force that centers a microtubule aster (24–27). Finally, active force generation at cell surface anchor sites can exert cortical pulling forces upon astral microtubules and spindle via the dynein-associated protein LIN-5/NuMA (28, 29). Dynein-dependent cortical pulling forces at tricellular junctions in *Drosophila* epithelia, and at retraction fibers in cultured cells, facilitate alignment of the cell division axis with the interphase long axis (6, 7). In addition, the LIN-5/NuMA complex also orchestrates spindle positioning and elongation in the *Caenorhabditis elegans* zygote (30–33). However, exerting cortical pulling forces onto astral microtubules gives rise to a tug-of-war scenario that can lead to unstable behaviors and even oscillations, which makes it challenging to robustly define positions and orientations (34–36). In conclusion, all three above mechanisms of microtubule-based force generation can contribute to Hertwig’s long axis rule (5–7, 14, 16, 21, 37, 38).

## Results

**Fig. 1.**
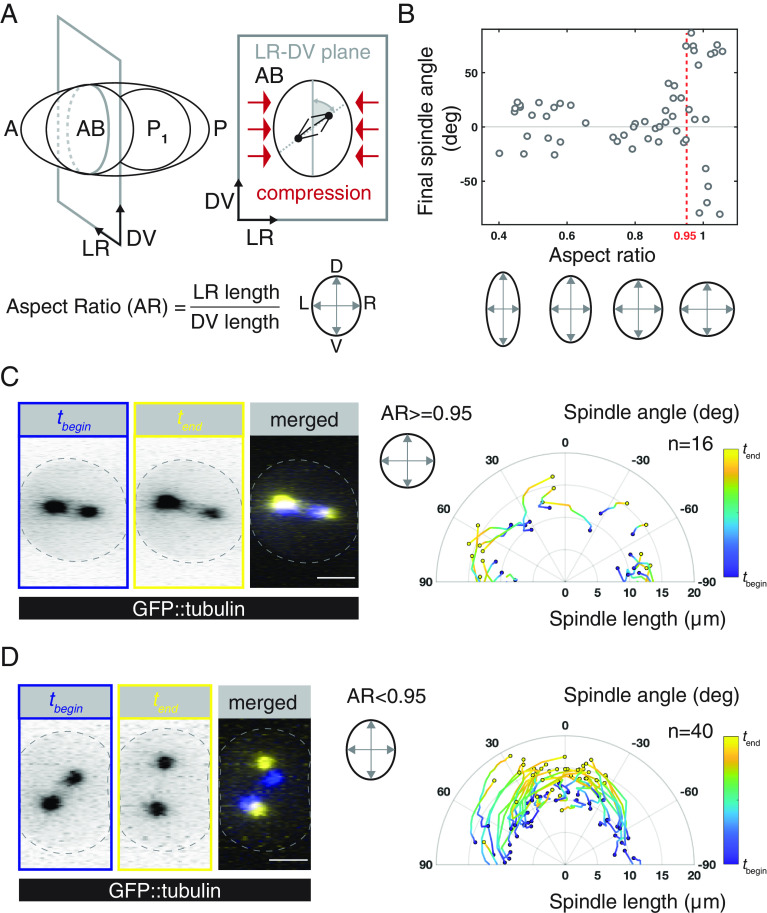
Hertwig’s long axis rule is executed in the AB cell. (*A*) *Left*: Schematic of a *C. elegans* two-cell embryo, anterior (A) and posterior (P) are indicated. *Right*: In utero, the embryo is compressed orthogonal to the anteroposterior (AP) axis such that the AB cell has a long and a short axis in the dorsoventral-left-right (DV-LR) plane. (*B*) Final angle between the mitotic spindle and the long axis in the DV-LR plane upon different compression strengths. Embryos were dissected and subjected to various degrees of compression, resulting in aspect ratios between 0.4 and 1.1. (*C* and *D*) *Left* panels: Example of an uncompressed (AR > 0.95, *C*) and compressed embryo (AR > 0.95, *D*), producing GFP-tubulin, at the beginning of anaphase (*t_begin_*, blue) and at the end (*t_end_*, yellow) viewed in the DV-LR plane. The end point is defined by the starting time point of the cell division skew in the AP-DV plane (*SI Appendix*, Fig. S1). Dashed lines mark the cell outline, as defined by the Lifeact::mKate2 signal (Movies S1 and S2). *Right* panels: Time evolution of spindle length (pole-to-pole distance) and angle with the long axis in uncompressed (*C*) and compressed (*D*) embryos, plotted in polar coordinates. Traces represent individual embryos. Time is normalized and subsequently color coded in the same manner throughout the manuscript. For uncompressed embryos, in which there is no long axis, the angle with the imaging plane was reported. (Scale bars: 10 μm.)

We here set out to investigate how Hertwig’s rule is executed in systems where cortical pulling forces onto astral microtubules are prevalent. To this end, we first study long axis finding in early *C. elegans* blastomeres that divide inside an ellipsoid eggshell (39). LIN-5/NuMA-dependent cortical pulling forces have been shown to orient mitotic spindles in early blastomere divisions (30–33, 40, 41). Since cell polarity cues can override Hertwig’s rule (9, 42, 43), we focus on the first symmetric cell division, which is the division of the unpolarized anterior blastomere in the two-cell embryo (Fig. 1*A*, AB cell). It has previously been shown that, guided by cell–cell contact with the posterior P_1_ cell (Fig. 1*A*), the AB cell initially sets up the mitotic spindle in the plane orthogonal to the AP axis (44–46) (the dorsal–ventral left–right, or DV-LR plane, Fig. 1*A*). The spindle remains in the DV-LR plane up until a cell rotation event at very late stages of cytokinesis (41, 44, 45, 47) (*SI Appendix*, Fig. S1, *Right*).

Early embryos are slightly compressed in utero (41, 48) such that the AB cell contains a long and a short axis in the DV-LR plane (41) (Fig. 1*A*). We first asked whether the AB cell divides along the long axis in this plane. We used spinning-disc confocal microscopy to image the mitotic spindle (GFP::tubulin) and the cell cortex (Lifeact::mKate2) of isolated embryos that were mounted under various degrees of compression (*Materials and Methods*). We measured the angle between the mitotic spindle and the long axis of the AB cell in the DV-LR plane (referred to as the spindle angle). While there is no preferred orientation in the absence of compression and for aspect ratios above ~0.95 (Fig. 1 *B* and *C* and Movie S1), the mitotic spindle is aligned with the long axis of the cell at the end of anaphase in embryos compressed to aspect ratios below ~0.95 (spindle angle: 4.4 ± 20.2°, mean ± SD throughout the text, n = 40; Fig. 1 *B* and *D* and Movie S2). Importantly, it was previously reported that the aspect ratio in utero is ~0.86 (41). Altogether, this demonstrates that, when compressed, the dividing AB cell follows Hertwig’s long axis rule. This is so even though the AB spindle is randomly oriented at the onset of cell division and during metaphase (Fig. 1*D*). Misaligned mitotic spindles then underwent a rotation such that, by the end of anaphase, spindles aligned with the long axis (Fig. 1 *B* and *D* and *SI Appendix*, Fig. S1 *A* and *B* and Movie S2). Notably, after long axis alignment in the DV-LR plane, the AB cell, including its mitotic spindle, tilts in the AP-DV plane. This phenomenon has been characterized previously (41), and we here focus on the long-axis alignment that occurs earlier during anaphase. Altogether, Hertwig’s rule is executed in the dividing AB cell via a spindle rotation.

**Fig. 2.**
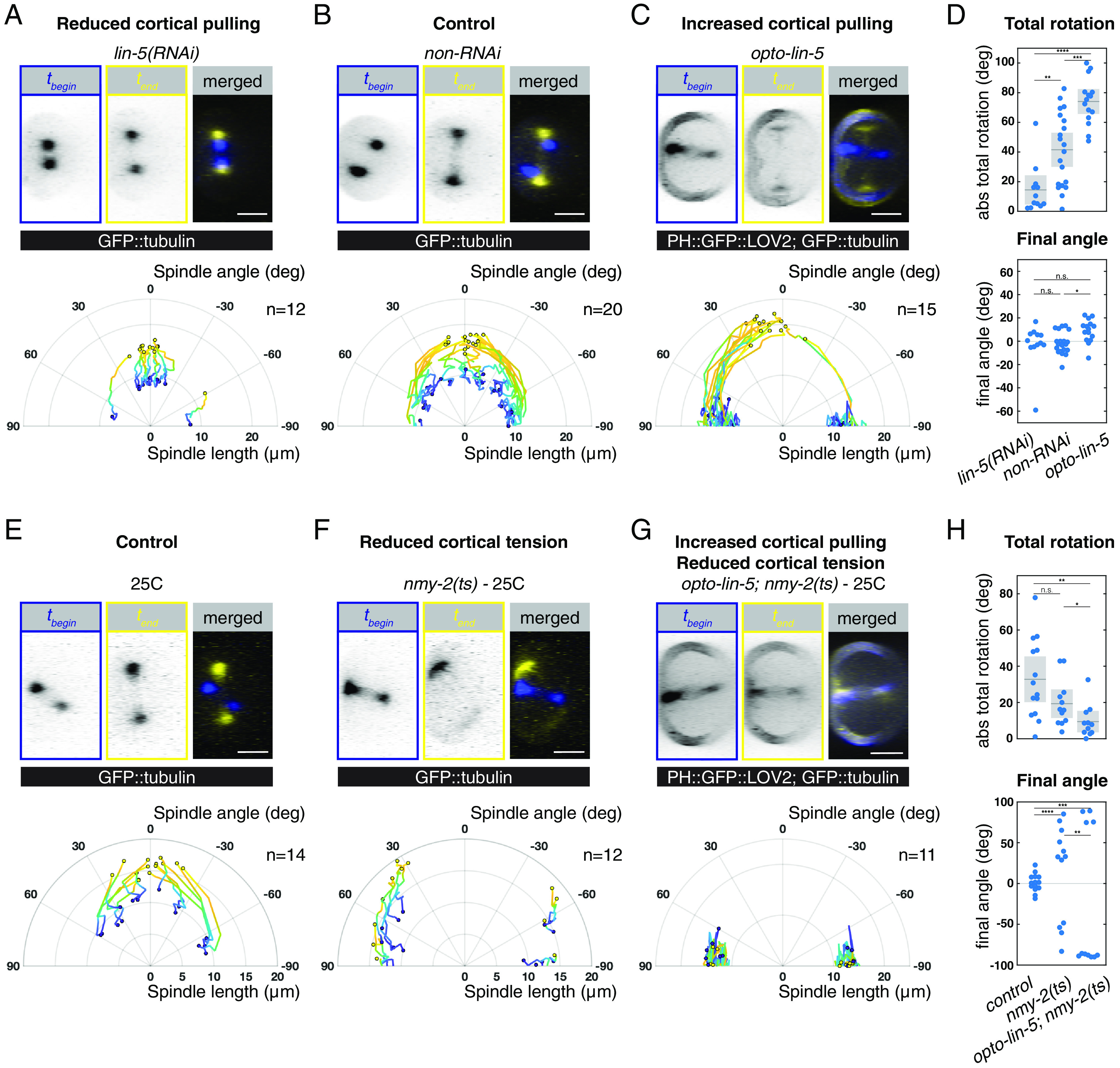
Long axis alignment in the AB cell is achieved by an NMY-2/Myosin-dependent spindle rotation. (*A*–*C*) Time evolution of spindle length and angle upon (*A*) decreased cortical pulling forces, *lin-5(RNAi)*, (*B*) control, *non-RNAi,* and (*C*) increased cortical pulling forces, *opto-lin-5*. (*D*) Total absolute angular movement of the spindle (*Top*) and final spindle angle with the long axis (*Bottom*). Data points represent individual embryos. Mean with 95% CI is indicated for the total rotation. Statistical tests: Wilcoxon rank-sum test for total angular movement and, because of the periodicity of the data, the Watson U^2^ test for final spindle angles. (*E*–*G*) Time evolution of spindle length and angle imaged at 25 °C in (*E*) control, (*F*) upon reduced cortical tension-*nmy-2(ts)* and (*G*) upon combined cortical tension reduction and increased cortical pulling forces—*opto-lin-5; nmy-2(ts)*. The GFP channel in *opto-lin-5* shows GFP::tubulin and PH::GFP::LOV2, which is a membrane-localized LOV2 domain necessary to recruit cytoplasmic LIN-5::ePDZ to the cortex (*Materials and Methods*). (*H*) Total absolute angular movement of the spindle (*Top*) and final spindle angle with the long axis (*Bottom*), as in (*D*). Note: As we still observe a rotation of the spindle in *nmy-2(ts)* embryos, the NMY-2/Myosin activity may not be fully perturbed at 25 °C, or an NMY-2/Myosin-independent pathway exists. The control conditions for *opto-lin-5; nmy-2(ts)* imaged at 25 °C are shown in *SI Appendix*, Fig. S4 *A* and *B*. n = number of embryos. (Scale bars: 10 μm.)

We next asked whether long axis alignment is driven by LIN-5/NuMA-dependent cortical pulling forces (28). To address this, we analyzed spindle rotation in the AB cell in compressed embryos upon *lin-5/NuMA(RNAi)* (30, 49). Cortical pulling forces were significantly reduced in this condition, as evidenced by a decreased spindle length and reduced transverse spindle pole fluctuations prior to anaphase (*SI Appendix*, Fig. S2). Interestingly, we find that the metaphase spindle in *lin-5/NuMA(RNAi)* embryos was already aligned with the long axis of the cell (initial spindle angle: −4 ± 33.3°, n = 12, see *SI Appendix*), and remained so during anaphase (final spindle angle: 0.2 ± 18.2°, n = 12, Fig. 2 *A* and *D* and Movie S3). This is in contrast to unperturbed embryos where the spindle was randomly oriented at metaphase and aligned later during anaphase (Fig. 2 *B* and *D* and Movie S4). Together, this indicates that cortical pulling forces counteract long-axis finding during metaphase.

If Dynein-mediated cortical pulling forces indeed counteract AB spindle long-axis alignment during metaphase, then upregulation of cortical LIN-5/NuMA should lead to metaphase AB spindle short-axis alignment. To test this, we made use of a previously described optogenetic tool, in which cortical LIN-5/NuMA levels are increased upon blue light-illumination (referred to as *opto-lin-5*), thereby increasing pulling forces on astral microtubules (40). We dissected embryos with minimal light exposure and kept them in the dark until 3 to 4 min after completion of the first cytokinesis. Thereafter, opto-LIN-5 was globally activated by 3D imaging of the GFP channel. We analyzed the localization of LIN-5/NuMA during metaphase in control and *opto-lin-5* embryos. In both conditions, we found that LIN-5/NuMA localizes symmetrically in the AB cell (*SI Appendix*, Fig. S3 *A–C* and Movie S5), in line with previous findings (31, 40). Moreover, global blue-light illumination in *opto-lin-5* embryos resulted in fast and uniform enrichment of LIN-5/NuMA at the cortex (*SI Appendix*, Fig. S3*C* and Movie S5). Together, this resulted in an increased spindle length and transverse spindle pole fluctuations prior to anaphase, indicative of elevated cortical pulling forces (*SI Appendix*, Fig. S2 and Movie S6). Finally, consistent with our expectation, increasing cortical LIN-5/NuMA levels leads to AB metaphase spindles that are aligned with the short axis (initial spindle angle: −85.2 ± 8.6°, n = 15, Fig. 2*C*), a phenotype opposite to that observed in *lin-5/NuMA(RNAi)* (Fig. 2*A*). However, this initial misalignment is corrected during anaphase via a spindle rotation (final spindle angle: −9 ± 9.5°, n = 15; Fig. 2 *C* and *D* and Movie S6), thereby ensuring Hertwig’s rule.

Since all our LIN-5/NuMA perturbations affected metaphase, but not anaphase long-axis alignment, we conclude that a LIN-5/NuMA-independent mechanism ensures Hertwig’s rule in this system. Taken together, Dynein-mediated cortical pulling forces favor short-axis alignment of the mitotic spindle during metaphase in the AB cell, which is corrected during anaphase by a LIN-5/NuMA-independent spindle rotation.

We next set out to identify the LIN-5/NuMA-independent mechanism of rotation for achieving long-axis spindle alignment. We note that the actomyosin cortex has also been implicated in Hertwig’s rule albeit with mechanisms that are not well understood (8, 28, 50–53). To test whether myosin-dependent force generation contributes to Hertwig’s rule in the AB cell, we perturb non-muscle myosin II (hereafter referred to as NMY-2/Myosin), activity by making use of a temperature-sensitive *nmy-2/Myosin* mutant (*nmy-2(ne3409ts),* hereafter referred to as *nmy-2(ts)*) (54). This allele yields functional NMY-2/Myosin at the permissive temperature of 15 °C, and largely inactive NMY-2/Myosin at the restrictive temperature of 25 °C (54, 55). Embryos were kept at the permissive temperature until completion of the first cell division, then shifted to the restrictive temperature, followed by analysis of AB spindle dynamics in the DV-LR plane.

Acute inactivation of NMY-2/Myosin leads to spindles that tend to align with the short axis in the DV-LR plane during metaphase (Fig. 2 *E* and *F*). This is expected given a reported inhibitory effect of the actomyosin cortex on cortical pulling forces (56–58): An acute loss of NMY-2/Myosin activity should therefore lead to increased cortical pulling forces and short axis alignment, as was indeed observed (Fig. 2*F*). Strikingly however, the rotation of the spindle during anaphase was impaired upon loss of NMY-2/Myosin activity (19.3 ± 12.7 degrees of rotation in *nmy-2(ts)* for embryos where the initial spindle angle was between 30 and 90°, Movie S8, compared to 47.2 ± 17 in unperturbed control embryos, Movie S7), and the mitotic spindle failed to align with the long axis at the end of anaphase upon acute NMY-2/Myosin inactivation (final spindle angle: 68 ± 36°, n = 12; Fig. 2 *F* and *H* and Movie S8; note that spindle rotation was not fully impaired in the *nmy-2(ts)*). This provides evidence that actomyosin drives the rotation during anaphase that ensures Hertwig’s rule in the AB cell. We further tested this possibility by elevating cortical pulling forces when NMY-2/Myosin activity was impaired. To this end, we analyzed *opto-lin-5; nmy-2(ts)* embryos at restrictive temperature. As in *opto-lin-5* and *nmy-2(ts)* alone, the metaphase spindle was aligned with the short axis in *opto*-*lin-5*; *nmy-2(ts)* embryos imaged at 25 °C (initial spindle angle: 85.3 ± 12.6 degrees, n = 11; Fig. 2*G*), but now remained so throughout anaphase (final spindle angle: 88.3 ± 6.4°, n = 11, Fig. 2 *G* and *H* and *SI Appendix*, Fig. S4 and Movie S9). Note that under these conditions the final spindle length was reduced in comparison to the unperturbed control, probably due to a lack of space (Movie S9, *Right* panel, *SI Appendix*, Fig. S4*C*). Together, we conclude that LIN-5/NuMA-mediated cortical pulling forces and NMY-2/Myosin-driven contractility act antagonistically: LIN-5/NuMA-dependent cortical pulling forces favor spindle short-axis alignment, which is then corrected into spindle long-axis alignment via an NMY-2/Myosin-dependent rotation.

**Fig. 3.**
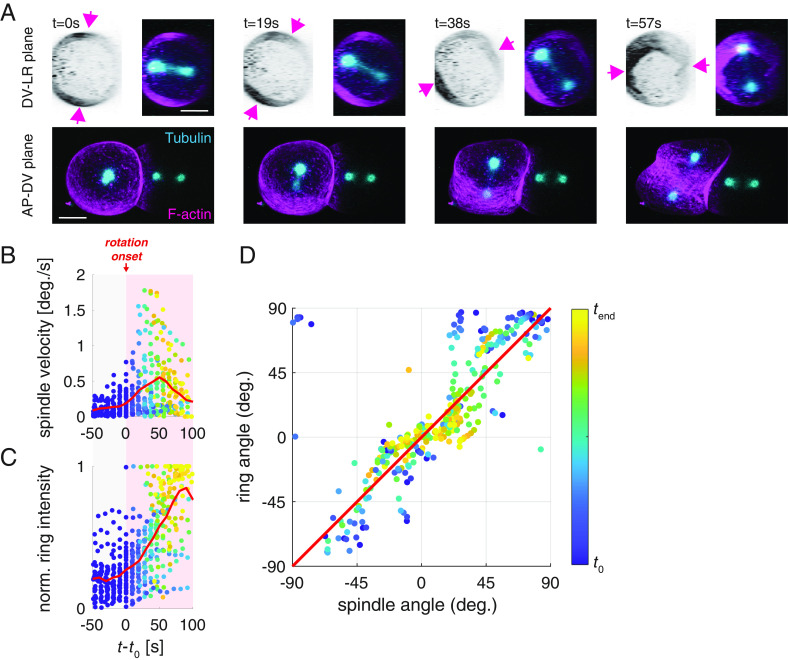
The mitotic spindle and cytokinetic ring rotate together, and this coincides with cytokinetic ring formation. (*A*) Still images of a two-cell embryo producing GFP::tubulin (cyan) and Lifeact::mKate2 (magenta and inverted) viewed in the DV-LR plane (*Top*) and AP-DV plane (*Bottom*). Arrows mark the position of the cytokinetic ring. (Scale bar: 10 μm.) (*B*) Time evolution of the angular velocity of the mitotic spindle and (*C*) the normalized F-actin intensity in the ring (*SI Appendix*, Eq. **S3**). *t_0_* (=*t_begin_*) is defined as the rotation onset. Red lines show the mean. As the angular velocity is noisy, data points in (*B*) represent a moving average with 45 s as sliding window. (*D*) Angle of the spindle and ring during the rotation plotted over time. Note that the angles for spindle and ring are with respect to the long axis and short axis, respectively. The red diagonal is a guide to the eye to indicate the similarity of spindle and ring orientation. Color-coded data points in (*B*–*D*) represent individual measurements at respective time points.

How could NMY-2/Myosin drive the rotation that leads to long axis alignment of the spindle? One possibility is that the constriction of the cytokinetic ring could drive a rotation when the overall shape of the cell is fixed by the eggshell (59). To determine whether the cytokinetic ring contributes to long axis alignment in the AB cell, we first investigate the temporal relationship between spindle rotation and cytokinetic ring formation in the AB cell. We perform simultaneous imaging of the actomyosin cortex and the mitotic spindle and find that spindle rotation is concomitant with the formation and onset of cytokinetic ring ingression (Fig. 3 *A–C* and *SI Appendix*, Fig. S5). This analysis also reveals that the ring and the spindle rotate together (Fig. 3*D* and *SI Appendix*, Fig. S5 and Movie S2), suggesting that AB spindle rotation is achieved by rotating the entire AB cell, including all of its intracellular components (*SI Appendix*, Fig. S6*A*). Furthermore, the AB cell is in direct contact with its neighboring P_1_ cell, which also rotates at the same time (*SI Appendix*, Fig. S6*B* and Movie S10) indicating that NMY-2/Myosin activity in the AB cell drives both a whole-cell and whole-embryo rotation. Together, this shows that the whole embryo is essentially free to rotate inside the eggshell, consistent with earlier observations (47, 48, 60, 61).

**Fig. 4.**
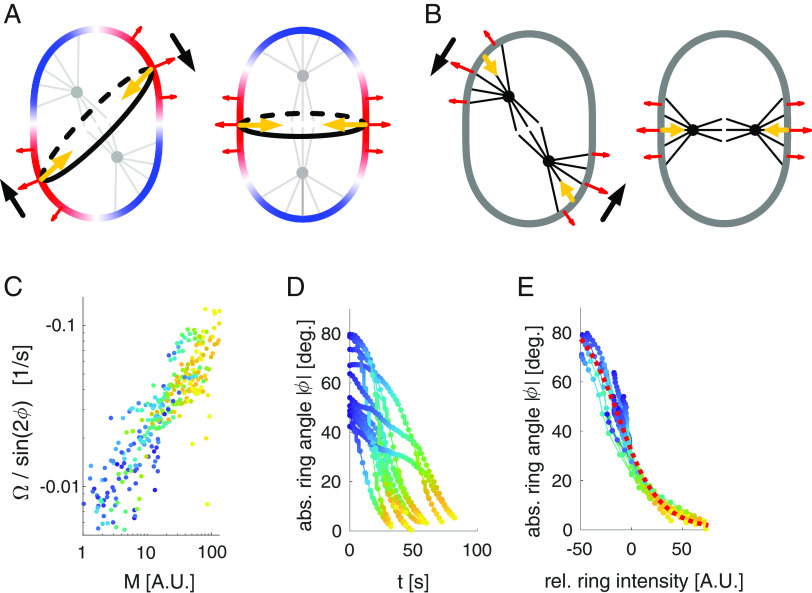
Physical model of long axis alignment driven by the emerging cytokinetic ring. (*A*) Schematic of the AB cell in the DV-LR plane: Rigid eggshell enforces shape of the embryo by exerting forces (red arrows) normal to the embryo surface that balance forces resulting from active surface tension in the cell cortex (colored contour). The cytokinetic ring (black contour) is a region of high tension (red) relative to the cell poles (blue), where astral microtubules (gray) inhibit actomyosin. This results in normal forces driving ring ingression and pole expansion that are balanced by the eggshell. When the ring is not aligned perpendicular to the long axis (*Left*) a torque arises driving a rotation (black arrows) that aligns the ring perpendicular to the long axis and the spindle with the long axis. (*B*) Same as in (*A*) but for a scenario where normal forces are primarily due to cortical pulling by astral microtubules. In such a scenario, the cell poles are pulled inward, resulting in alignment of the spindle axis with the short axis of the cell. (*C*–*E*) Quantitative analysis of the dynamics of the angle *ϕ* between the ring and the short axis and the NMY-2 concentration M in the ring. (*C*) Scatter plot of effective force driving alignment determined from the speed of cortical rotation Ω and the ring angle *ϕ* vs. NMY-2 ring intensity M (*SI Appendix*, Eq. **S7**). We observe a striking correlation (rho = −0.8, *P*-value < 1e−3). (*D*) Trajectories of the absolute ring angle |*ϕ*|. (*E*) Same trajectories as in (*D*) but plotted vs. the change in ring intensity relative to the ring intensity at |*ϕ*| = 30°. Trajectories collapse onto an exponential decay of the tangent of *ϕ* as predicted by our model for a linear relationship between active tension and NMY-2 concentration.

How can cell-internal stresses such as the ones generated by NMY-2/Myosin drive a rotation of the entire two-cell embryo? For such a rigid body–like rotation to occur, internal stresses must generate a torque against the stationary eggshell. We consider a scenario where cytoplasmic pressure pushes the cortex and cell membrane against the eggshell, such that the overall shape of the embryo is fixed by the shape of the rigid eggshell, but the embryo is free to rotate within the confining shell (47, 48, 60, 61). During anaphase, NMY-2/Myosin-driven active tension in the cytokinetic ring generates inward forces that will later drive its ingression (62–65). These inward forces are balanced by outward forces exerted by the eggshell, perpendicular to the surface of the embryo. When the ring of the AB cell is not aligned with the short or long axis of the embryo in the DV-LR plane, the resultant pattern of surface normal forces yields a torque exerted onto the embryo from the eggshell, causing the entire embryo to rotate inside the eggshell (Fig. 4*A* and *SI Appendix*, *Supplementary Notes*). Notably, this rotation of the entire embryo will rotate the cytokinetic ring away from the long axis and aligns it with the short axis, and thereby align the spindle with the long axis in the DV-LR plane. Importantly, the same physical argument can be applied to the effects of Dynein-mediated cortical pulling forces by astral microtubules giving rise to inward forces at the cell poles, where astral microtubules are in contact with the cell surface (Fig. 4*B*). However, the torque associated with such forces is expected to drive a rotation of the spindle toward short axis alignment (Fig. 4*B* and *SI Appendix*, *Supplementary Notes*). This is consistent with our observations in embryos with elevated cortical pulling and reduced Myosin activity (*opto*-*lin-5*; *nmy-2(ts),* Fig. 2*G*). Together, this suggests that for a cell whose shape is constrained but free to rotate inside the eggshell, cytokinetic ring tension can drive a cell rotation to ensure Hertwig’s rule.

To quantitatively test our physical model, we recorded 14 divisions of the AB cell at high time resolution (dt = 2 s) via spinning-disc microscopy of endogenously labeled NMY-2/Myosin::GFP (Movie S10). Our general theory predicts that the speed of rotation Ω is determined by the angle ϕ between the ring and the short axis and the active tension T generated in the ring according to Ω=-αT sin 2ϕ, where the coefficient α depends on the aspect ratio of the embryo in the DV-LR plane, the friction between embryo and eggshell, and an effective viscosity of the embryo. Fig. 4*C* reveals that Ω/ sin 2ϕ increases monotonously with the intensity of NMY-2/Myosin M in the ring, as expected for a linear or saturating relationship between the amount of NMY-2/Myosin M and active tension T in the cytokinetic ring. Furthermore, we find that the amount of NMY-2/Myosin M increases exponentially with time during ring formation (*SI Appendix*, Fig. S7*E*), leading to a prediction of the model where the tangent of phi decreases exponentially with increasing M (*SI Appendix*, *Supplementary Notes*). Indeed, we find that the experimentally measured trajectories of phi collapse onto such a curve when plotting phi vs. M (Fig. 4 *D* and *E*). Taken together, these results are consistent with a scenario where Hertwig’s rule in the AB cell is facilitated by NMY-2/Myosin-dependent force generation in the emerging cytokinetic ring, which drives a whole-cell rotation in the case of misalignment.

**Fig. 5.**
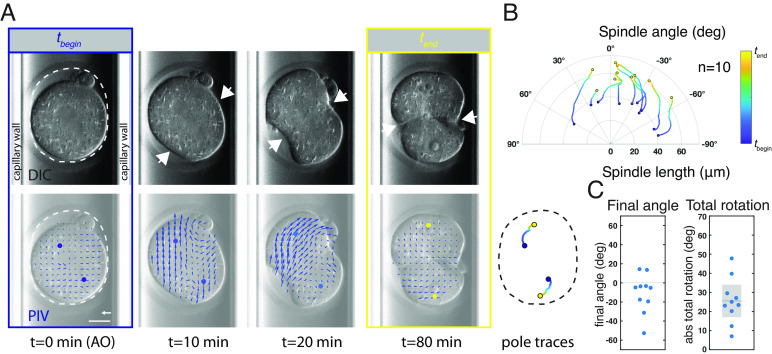
Hertwig’s rule execution in slightly compressed mouse embryos coincides with ring ingression. (*A*) *Top* panels: Example of a mildly compressed mouse zygote, imaged using Differential Interference Contrast (DIC) microscopy, undergoing cell division inside a glass capillary. For mild compression, embryos inside a capillary with inner diameter between 80 and 90 µm were used. The dashed line indicates the shape of the zona pellucida. *Left* images show anaphase onset (*t_begin_*, blue), and *Right* images show the zygote upon completion of cytokinesis (*t_end_*, yellow). The embryo is slightly compressed such that the zona pellucida has a long and a short axis. During metaphase, the mitotic spindle is not aligned with the long axis of the zona pellucida, but during anaphase, it rotates toward the long axis. Arrows mark the cytokinetic ring. *Bottom* panels: Still images overlaid with the cytoplasmic flow field as measured by PIV. Spindle poles, marked with filled circles, were manually identified at anaphase onset (*Left*), and their positions were inferred by using the interpolated local flow field (*Materials and Methods*). Spindle pole time traces of the displayed zygote are shown on the lower right. (*B*) Time evolution of spindle length (pole-to-pole distance) and angle with the long axis in mildly compressed mouse zygotes, plotted in polar coordinates. During ring ingression, the spindle rotates toward long axis alignment. In 2 out of 10 embryos, long axis alignment was not finished upon completion of cytokinesis. In these embryos, a slower rotation into long axis alignment occurred in the two-cell embryo, see Movie S14. (*C*) Final angle with the long axis and total absolute spindle rotation in degrees. Each data point represents an individual embryo. Mean with 95% CI is indicated for the total rotation. n = number of embryos. AO = anaphase onset. PIV vector scale bar: 1 μm/min. (Scale bar: 20 μm.)

Given that early embryonic cells during holoblastic cleavages are not reported to be anchored to the confining shell, we argue that they are often free to rotate. This suggests that the actomyosin-dependent mechanism of long-axis alignment we have identified is general in early cell divisions. To evaluate this, we next studied the first division in mouse embryos, in which it has previously been shown that Hertwig’s rule applies (11). Because the mitotic spindle in mouse zygotes is anastral (66, 67), we hypothesize that long axis alignment occurs via contractility in the cytokinetic ring, akin to the *C. elegans* AB cell. To study this, we deformed mouse zygotes into an elliptical shape using glass pipettes (Movies S11–S13) followed by time-lapse imaging using Differential Interference Microcopy (DIC). We find that the first cell division in 8 out of 17 embryos inside capillaries was slightly asymmetric (*SI Appendix*, Fig. S9 and *Supplementary Notes*). Importantly, we found that cell division indeed aligns with the long axis in both mildly and strongly compressed embryos (n = 15 embryos, Fig. 5 and *SI Appendix*, Fig. S8 and Movies S12 and S13).

We next asked whether mouse zygotes undergo a whole-cell rotation during anaphase, to align the division axis with the long axis. To address this, we measured the length (pole-to-pole distance) and the angle of the mitotic spindle with the compression-induced long axis during mitotic progression. We extracted spindle pole positions from our DIC recordings by first manually identifying both spindle poles during metaphase, and subsequently inferring their movement from the cytoplasmic flow field (*Materials and Methods*). In strongly compressed zygotes the metaphase spindle was already aligned with the long axis and remained so during anaphase (n = 5 embryos, *SI Appendix*, Fig. S8 *C* and *D* and Movie 13). However, in mildly compressed zygotes, metaphase spindle orientation was unaffected by compression (n = 10 embryos, Fig. 5 *A* and *B* and Movie S12). Importantly, in all cases where the metaphase spindle was misaligned with the long axis of the mildly compressed egg, the zygote underwent a rotation at the time of cleavage furrow ingression. This rotated the entire cell, including the cytoplasm and its constituents, thereby aligning the mitotic spindle with the long axis of the cell (Fig. 5 *A–C* and *SI Appendix*, Fig. S8*E* and Movie 12). The mitotic spindle in mouse zygotes is anastral and without direct microtubule connections to the cortical surface (66, 67). Hence, our results are compatible with a mechanism where the whole-cell rotation, through hydrodynamic coupling in a viscous cytoplasm, rotates the entire cell together with its content, which includes the mitotic spindle. We note that the rotation in mouse zygotes coincided with ring ingression and often continued up until ring closure. This is different from the scenario in the *C. elegans* AB cell, where rotation started during ring formation and finished prior to discernible ring ingression (*SI Appendix*, *Supplementary Notes*). Taken together, experiments with slightly compressed mouse zygotes reveal that, also here, the entire cell rotates as the cytokinetic ring constricts, thereby aligning cell division with the geometric long axis. These results suggest that the cytokinetic ring can execute Hertwig’s rule in holoblastic divisions of evolutionary distant species.

## Discussion

In summary, we suggest the following mechanism for long axis spindle alignment: In scenarios where cell shapes are constrained and where cells are free to rotate, long axis alignment is a consequence of active tension generation in the cytokinetic ring, which corrects any previous misalignment of the mitotic spindle. Importantly, when cortical pulling forces onto astral microtubules are not dominant, spindles may already align with the long axis of the cell during metaphase, via cytoplasmic pulling forces (5, 14, 16, 18–21) or via pushing (22, 24–27) (Fig. 2*A*). Active tension generation in the cytokinetic ring will maintain this alignment. In cases where cortical pulling forces are dominant, spindles first align with the short axis of the cell. However, since the cytokinetic ring establishes in a plane that is orthogonal to the spindle axis, the ring will now not be aligned with the short axes of the cell. Myosin-driven tension in the cytokinetic ring will then drive a whole cell (or whole embryo) rotation that aligns the ring with the short axis, thereby leading to alignment of the spindle with the long axis. Shapes of cells and patterns of surface forces can be more complex than considered here, but the principle of undergoing a rotation to align a pattern of actomyosin-generated surface forces with cell shape remains general (*SI Appendix*, *Supplementary Notes*). We suggest that this actomyosin-dependent mechanism of executing Hertwig’s rule is an important contributor to spindle positioning and cell division orientation during early development.

## Materials and Methods

### Animal Strains and Culturing.

*C. elegans* strains were cultured using standard culture conditions (68) and maintained at 20 °C, apart from strains carrying *nmy-2(ne3409ts)*, which were maintained at 15 °C. For a list of *C. elegans* alleles and transgenes used in this study, see *SI Appendix*. Mouse embryos were from a CD-1(ICR) outbred background in natural mating (without hormone indication). The mice were kept under pathogen-free conditions (SOPF) and were provided with ad libidum feed and water. The animal room has a day/night rhythm of 12/12 h from 6 AM to 6 PM. Mating is scheduled in the early afternoon around 2 PM.

### Microscopy Setups.

All *C. elegans* imaging was done using spinning disc microscopy. Apart from the LIN-5 localization experiments, imaging was done on a Zeiss Axio Observer Z1 inverted microscope equipped with a Yokogawa CSU-X1 scan head, a C-Apochromat 63×/1.2 NA W objective, a Hamamatsu ORCA-flash 4.0 camera, 488 and 560 lasers, and operated by Micromanager software. The LIN-5 localization experiments were done on a Nikon eclipse Ti2 inverted microscope with a Yokogawa CSU-W1 scan head, a CF Plan Apo VC 60×/1.2 NA W objective, Teledyne Photometrics Prime BSI camera, 488 and 561 laser, and operated by Nikon elements software. Imaging was performed at room temperature (22 to 23 °C) apart from the experiments using the *nmy-2(ne3409ts)* and the accompanying controls. For these experiments, the Cherry temp microscopy stage (Cherry Biotech) was used to switch between 15 and 25 °C. Mouse DIC imaging was done using a Zeiss Axio Observer Z1 inverted stand equipped with DIC optics, an NA0.55 WD 26 mm condenser, a 40×/NA1.1 W objective, and a Zeiss Axiocam 705 MRm monochrome CCD camera. Temperature (37 °C), CO2 levels (5%), and humidity were maintained using a stage top incubator. An objective heater was used to prevent heat dissipation.

### RNA Interference.

RNAi treatment was performed by feeding as previously described (69). Briefly, NGM agar plates containing 1 mM isopropyl-beta-D-thiogalactoside and 50 μg/mL ampicillin were seeded with bacteria expressing dsRNA targeting the gene of interest or containing empty vector (L4440). The L4440 control and *lin-5(RNAi)* clones were obtained from the Ahringer RNAi library (Source BioScience) (70). L4 larvae were grown on feeding RNAi plates at 20 °C for 25 to 27 h prior to embryo dissection.

### Image Preprocessing and Analysis.

Microscopy recordings were prepared and analyzed using Fiji (71) and Matlab, and data analysis was performed in Matlab. Particle Image Velocimetry was performed using an open source Matlab package (PIVlab) (72). For statistical analysis of periodic data, we used the Circular Statistics Toolbox in Matlab (73).

For details on sample preparation, image acquisition, and image analysis, see *SI Appendix*.

## Supplementary Material

Appendix 01 (PDF)

Movie S1. Dynamics of the mitotic spindle and actin cortex during AB cell division in an uncompressed embryo. Spinning disc imaging of an uncompressed embryo producing Lifeact::mKate2 and GFP::tubulin. 3D imaging was performed by making 30 *μ*m z-stacks (dz=1um) capturing the entire embryo. Subsequently, maximum intensity projections were made to visualize the DV-LR plane (left panels) and the AP-DV plane (right panels). Due to the lower axial resolution in the z-direction, pixels along the z-axis (LR axis) were interpolated on a grid with 0.1058 *μ*m spacing corresponding to the pixel size in the x- and y-axis (AP and DV respectively). Top panels show inverted GFP::tubulin channel alone and bottom panels show the merged Lifeact-mKate2 and GFP::tubulin channels.

Movie S2. Dynamics of the mitotic spindle and cortex during AB cell division in a compressed embryo. Spinning disc imaging of a compressed embryo producing Lifeact::mKate2 and GFP::tubulin. 3D imaging was performed by making 25 *μ*m z-stacks (dz=1um) capturing the entire embryo. Rest is as in Movie 1.

Movie S3. Dynamics of the mitotic spindle during AB cell division in a compressed *lin-5(RNAi)* embryo viewed in the DV-LR plane. Movie shows GFP::tubulin signal in the AB cell projected onto the DV-LR plane in *lin-5(RNAi)* embryo producing GFP::tubulin and endogenously labeled LIN-5::ePDZ::mCherry (not visualized).

Movie S4. Dynamics of the mitotic spindle during AB cell division in a compressed L4440 control embryo viewed in the DV-LR plane. Movie shows GFP::tubulin signal in the AB cell projected onto the DV-LR plane in an L4440 control embryo producing GFP::tubulin and endogenously labeled LIN-5::ePDZ::mCherry (not visualized).

Movie S5. LIN-5 localization in the two-cell embryo during AB anaphase progression. Movie shows endogenously labeled LIN-5::ePDZ::mCherry in a (left) control embryo that shows initial long axis alignment, a (middle) control embryo that shows initial short axis alignment, and an (right) *opto-lin-5* embryo upon global blue-light illumination. Top row shows the embryo midplanes, bottom row shows the cortical planes. The first time frame in the *opto-lin-5* embryo (right) is captured prior to global blue-light illumination. From the second time frame onwards, LIN-5::ePDZ::mCherry displays strong cortical enrichment in the AB and P1 cell. See Fig. S3 and its caption for further details.

Movie S6. Dynamics of the mitotic spindle during AB cell division in a compressed *opto-lin-5* embryo viewed in the DV-LR plane. Movie shows GFP::tubulin signal in the AB cell projected onto the DV-LR plane in an *opto-lin-5* embryo producing GFP::tubulin, PH::GFP::LOV2 and endogenously labeled LIN-5::ePDZ::mCherry (not visualized).

Movie S7. Dynamics of the mitotic spindle during AB cell division in a compressed control embryo at 25C viewed in the DV-LR plane. Movie shows GFP::tubulin signal in the AB cell projected onto the DV-LR plane in a control embryo producing GFP::tubulin and Lifeact::mKate2 (not visualized).

Movie S8. Dynamics of the mitotic spindle during AB cell division in a compressed *nmy-2(ts)* embryo at 25C viewed in the DV-LR plane. Movie shows GFP::tubulin signal in the AB cell projected onto the DV-LR plane in an *nmy-2(ts)* embryo producing GFP::tubulin and Lifeact::mKate2 (not visualized).

Movie S9. Dynamics of the mitotic spindle during AB cell division in a compressed *opto-lin-5*; *nmy-2(ts)* embryo and its controls at 25C. Movie panels show GFP::tubulin signal in the AB cell projected onto the DV-LR plane in *opto-lin-5* (left), *nmy-2(ts)* (middle) and *opto-lin-5*; *nmy-2(ts)* (right) embryos imaged at 25C. Because expression levels of GFP::tubulin varied, the contrast in the movies was differently adjusted.

Movie S10. Cortical movements in the AB cell during whole embryo rotation viewed in the AP-DV plane. Top panel: Cortical rotation of an embryo producing endogenously labeled NMY-2::GFP, imaged using high time resolution (dt=2s). Only the cortical surface was imaged in the AP-DV plane and displayed as inverted color. Bottom panel: Same embryo with the flow field, measured using Particle Image Velocimetry (PIV), overlaid. Both the anterior AB cell and the neighboring P1 cell undergo a similar rotation within the stationary egg shell. Scale bar=10 *μ*m.

Movie S11. Uncompressed mouse zygote undergoing division inside a glass capillary. Differential Interference Contrast (DIC) movie of a non-compressed mouse zygote undergoing cell division inside a glass capillary, focussed on the central plane of the embryo. The cytoplasmic flow field in each timepoint, as measured by PIV, is overlaid (right side). The positions of the spindle poles were manually marked in the first time point at anaphase onset, and their approximate movement was subsequently inferred using the local cytoplasmic flow field. No reorientation of the cell division axis is observed.

Movie S12. Slightly compressed mouse zygote undergoing division inside a glass capillary. Differential Interference Contrast (DIC) movie of a slightly compressed mouse zygote undergoing cell division inside a glass capillary, focussed on the central plane of the embryo. The cytoplasmic flow field and the inferred spindle pole positions were overlaid as in Movie 11. The cell division axis reorients during anaphase in order to ensure Hertwig’s rule execution.

Movie S13. Strongly compressed mouse zygote undergoing division inside a glass capillary. Differential Interference Contrast (DIC) movie of a strongly compressed mouse zygote undergoing cell division inside a glass capillary, focussed on the central plane of the embryo. The cytoplasmic flow field and the inferred spindle pole positions were overlaid as in Movie 11. The cell division axis is already aligned along the long axis prior to anaphase and remains aligned.

Movie S14. Slightly compressed mouse zygote undergoing division inside a glass capillary. Differential Interference Contrast (DIC) movie of a slightly compressed mouse zygote undergoing cell division inside a glass capillary, focussed on the central plane of the embryo. The cytoplasmic flow field and the inferred spindle pole positions were overlaid as in Movie 11. This embryo undergoes a rotation towards alignment during ring ingression, but does not reach proper long axis alignment during cytokinesis. However, after cytokinesis the two-cell embryo continues a slow rotation into long axis alignment, presumably due to space constraints. We observed such behavior in 2 out of 10 mildly compressed embryos.

## Data Availability

Microscopy data have been deposited in https://edmond.mpdl.mpg.de/ (74).
