# Supplementary material for: A cytokinetic ring-driven cell rotation achieves Hertwig’s rule in early development
Source: Proc Natl Acad Sci U S A. 2024 Jun 13;121(25):e2318838121. doi: 10.1073/pnas.2318838121 (PMC11194556; doi:10.1073/pnas.2318838121)
Supplement: Supplementary file 1 — Appendix 01 (PDF) [file pnas.2318838121.sapp.pdf]

Supporting Information for

A cytokinetic ring-driven cell rotation achieves Hertwig's rule in early development

TC Middelkoop, J Neipel, CE Cornell, R Naumann, LG Pimpale, F Jülicher, SW Grill

T.C.M. (teije.middelkoop@img.cas.cz), F.J. (julicher@pks.mpg.de) and S.W.G. (grill@mpi-cbg.de)

This PDF file includes:

Supporting text
Figs. S1 to S10
Legends for Movies S1 to S14
SI References

Other supporting materials for this manuscript include the following:

Movies S1 to S14

Supporting Information Text

Supplementary methods

Experimental Methods.

Animal strains and culturing. *C. elegans* alleles and transgenes used in this study are: LGI, *nmy-2(ne3409ts)* (1), *nmy-2(cp8[nmy-2::gfp])*(2), LGII, *lin-5(he330[lin-5::glo-epdz::mcherry])*(3), LGV, *ruIs57[GFP::tubulin]*, LGIV, *cxTi10816(he259[ph::co-egfp::co-lov])*(3), LG unknown, *gesIs003[lifeact::mkate2]*(4). Mouse embryos were from a CD-1(ICR) outcrossed background in natural mating (without hormone indication).

C. elegans mounting and image acquisition. Worm embryos were dissected from young adults and mounted in M9. For mild compressions worms were mounted on 2% agarose pads. For stronger compressions, embryos were mounted in buffer containing 10 or 15 µm polystyrene spacer beads (Polysciences). Subsequently a glass slide was lowered over it such that the embryos were confined between the cover glass and the glass slide. The samples were sealed using valap to prevent evaporation of the buffer during imaging. In order to obtain uncompressed embryos, embryos were mounted in M9 on a coverslip coated with poly-L-lysin. Playing clay was fixed at the corners of the coverslip in order to achieve excess spacing.

For dual-color imaging of Lifeact-mKate2 and GFP-tubulin (control, non-RNAi and embryos carrying *nmy-2(ne3409ts)* without *opto-lin-5*) embryos were imaged using a 488 nm and 560 nm laser with 9-11 second time intervals. At each timepoint a z-stack was made of 26 slices (compressed embryos) or 31 slices (uncompressed embryos) with 1 μm spacing. For single color imaging of GFP-tubulin, embryos were imaged with 8 second time intervals (*lin-5(RNAi)*, *opto-lin-5* and accompanying controls) or 10 second time intervals (*opto-lin-5; nmy-2(ne3409ts)* and accompanying controls). At each timepoint a z-stack was made of 26 slices with 1 μm spacing. For the experiments using the *nmy-2(ne3409ts)* allele and the accompanying controls, embryos were kept at 15C until 3-4 min after completion of the first cytokinesis. Thereafter, the temperature was switched to 25C, followed by the start of image acquisition.

For imaging of the actomyosin cortex with high time resolution (dt=2 seconds), embryos producing endogenously labeled NMY-2::GFP (*nmy-2(cp8[nmy-2::gfp])*) were imaged using a 488 nm laser. Every time point (dt=2 sec) 2 focal planes with a z-spacing of 1 μm were recorded, as well as a midplane slice 10 μm above the cortical plane. For analysis a maximum intensity projection was made of the 2 cortical slices. Before the time lapse acquisition, a single z-stack was recorded of 31 slices with 1 μm spacing to extract the embryo outline in the DV-LR plane.

For imaging the endogenously tagged LIN-5::ePDZ::mCherry, control and *opto-lin-5* embryos were imaged using a 561 nm laser. Every time point (dt=10 sec) 2 focal planes with a z-spacing of 1 μm were recorded at the cortex, as well as a midplane slice 10 μm above the cortical plane. For visualization of the cortical plane, a maximum intensity projection was made of the 2 cortical slices. To achieve global blue-light illumination in *opto-lin-5* embryos we imaged the GFP channel both in the midplane and the cortical plane using the 488 nm laser. This blue-light illumination was started from the second time point onwards.

Mouse zygote mounting and image acquisition. For mouse zygote imaging, mouse cumulus complexes, including zygotes, were isolated from the oviduct of donor animals after a plug check at 8:00 am. With the help of the enzyme hyaluronidase, zygotes were washed out of the cumulus complex using M2 medium and selected for the presence of pronuclei. Before loading the embryos, predetermined breaking points were added to the hand-drawn glass capillaries with a ceramic cutter. This allowed the capillaries to be broken after loading without losing the embryos. Thereafter, zygotes were placed in glass capillaries (Hirschmann Ringcaps 50µl, handpulled) using a mouth pipette. These were then transferred to a 200µl droplet of KSOM media in dishes for incubation and microscopy (MatTek Corporation P35G-0.170-14-C), overlaid with Paraffin (Sigma-Aldrich, 1.07160.1000), and incubated and imaged at 37 degrees with 5% CO2. To generate the time-lapse recordings, every 60 seconds a 60-um z-stack with 2 μm z-spacing was acquired. Subsequently, for every time point the midplane z-slice was manually extracted.

For the quantifications of cell division asymmetry, still images of the 2-cell embryos were used. As a control, still images of 2-cell embryos that developed outside of a capillary were used. Notably, no time lapse imaging was performed on these control embryos. To determine whether mouse embryos can develop until later stages inside glass capillaries, we followed development of 8 embryos inside glass capillaries until the blastula stage by acquiring still images every 24 hrs in a separate experiment.

Image pre-processing and analysis of *C. elegans* recordings.

For visualization and analysis of the DV-LR plane, the image stacks were first rotated to align the AP axis horizontally. Images from the stacks were then cropped in the AP-DV plane to a 150-pixel wide region (15.87 μm) that spans the central part of the AB cell. Subsequently, a projection along the z axis, i.e. the AP axis, was made in every timepoint and pixel values were interpolated on a grid with 0.1058 μm spacing (corresponding to the pixel size in the x-y plane, i.e. the AP-DV plane) using cubic interpolation.

Spindle length (pole-to-pole distance) and angle with the DV axis were extracted by manually tracking the spindle poles, using the GFP-tubulin channel, in the DV-LR plane. To compute averages and standard deviations of the spindle orientation across embryos, we first computed an average phase factor corresponding to an average nematic order parameter as

$$Q_{\mathrm{av}} = \left\langle\, e^{2i\phi} \,\right\rangle \qquad [1]$$

with ϕ being the spindle angle and i the imaginary unit. The average angle ϕ_{av} and its (circular) standard deviation ϕ_{std} were then computed as

$$\phi_{\mathrm{av}} = \frac{1}{2}\arg\, Q_{\mathrm{av}}, \quad \phi_{\mathrm{std}} = \sqrt{-\log|Q_{\mathrm{av}}|/2} \qquad [2]$$

The AB cell outline in the DV-LR plane was extracted from the Lifeact-mKate channel or the NMY-2::GFP channel by applying segmentation using an intensity threshold. Because Lifeact-mKate levels varied among embryos the segmentation was manually supervised by varying the intensity threshold. Aspect ratios in the DV-LR plane were derived from these outlines by dividing the LR distance by the DV distance.

To extract the position of the cytokinetic ring from the Lifeact-mKate channel, a maximum intensity projection was first made of the two z-slices (dz=1 μm), closest to the imaging objective, that were cropped to a 30-pixel wide region centered at the center of the AB cell in the AP-DV plane. For every row of pixel values, the mean pixel values were extracted, yielding a vertical line of mean pixel values along the DV axis in each time point. Subsequently, these were concatenated to make kymographs from which the intensity of the cytokinetic ring was extracted. In addition, together with the AB cell outline in the DV-LR plane, this kymograph was used to extract the orientation of the cytokinetic ring in the DV-LR plane (see below).

The end point of the AB rotation was defined as the onset of the cell division skew on the AP-DV plane (Fig S1). For embryos producing a cortical marker (lifeact-mKate2 or NMY-2::GFP) the onset of rotation was defined as the first time point in which rotation was observed. For embryos without a cortical marker, the time point 160 seconds prior to the end point was taken as the first time point of analysis.

In order to determine the orientation of the ring, we assigned an azimuthal angle to each pixel of the outline of the cortex in the DV-LR plane. To do so, we used the center of mass $r_0 = (x_0, y_0) = \langle (x_j, y_j) \rangle$, determined from the positions $r_j = (x_j, y_j)$ of the pixels belonging to the cortex. Here x- and y-axis correspond to the axes in the LR-DV plane that are perpendicular and parallel to the imaging plane respectively. Due to compression perpendicular to the imaging plane the x (y)-axis corresponds to the short (long) axis in the LR-DV plane. The azimuthal angle θ_j for each pixel j in the LR-DV plane was then determined using

$$(x_j - x_0) + i\,(y_j - y_0) = r_j exp\,(i\theta_j) \qquad [3]$$

Where i is the imaginary unit and $r_j > 0$. With this we fitted the function

$$f\,(\theta) = f_0 + f_1 \cos 2\theta + f_2 \sin 2\theta \qquad [4]$$

to each time-point of the kymograph consisting of measured intensities I_j of LifeAct-mKate in the cortical plane. For each time point, we define the magnitude or ring intensity M and angle ϕ of the emerging cytokinetic ring as

$$f_1 + i f_2 = M \exp\,(2i\phi) \qquad [5]$$

where $M > 0$, such that $\phi = 0$ corresponds to a ring aligned with the x-axis, i.e. the short axis in the LR-DV plane (See Fig. S5A for an example). In Fig. 3C, the ring intensity M was normalized using the maximum value of M within the time interval of rotation for each embryo.

For quantifying movements of the actomyosin cortex in the AB cell with high time resolution (dt=2s), Particle Image Velocimetry (PIV) was performed using an open source Matlab package (PIVlab)(5) A 3-step PIV with a final box size of 36x36 pixels, on a grid with 18 pixel spacing (pixel size 0.1058 μm), was performed using a manually generated mask that segments the cortex of the AB cell in the AP-DV plane. The obtained flow fields were rotated to align the anteroposterior axis horizontally (anterior left, posterior right). Subsequently, velocity vectors at the anterior and posterior-most 15% of the AB cell mask were excluded. Of the remaining velocity vectors, the mean of the components perpendicular to the AP axis (DV velocity) was computed every time point. To visualize the movements of the AB cell together with the P1 cell, PIV was performed, with the same settings, on a movie rotated such that the AP axis is horizontally oriented. The analysis was done using a manually generated mask that segments the cortical of both the AB cell and the P1 cell. Cortical flow vectors were averaged over the time period of the rotation, to obtain the mean flow field.

To extract the position of the cytokinetic ring, and the ring intensity over time, from the cortical NMY-2::GFP signal, movies were first rotated to align the AP axis horizontally. Subsequently, a 90-pixel wide region of interest, covering the center part of the AB cell in the AP-DV plane, was used to make the kymograph. For every row of pixel values, the mean pixel values were extracted, yielding a vertical line of mean pixel values along the DV axis in each time point. Subsequently, these were horizontally concatenated to make kymographs from which the position and intensity of the cytokinetic ring were extracted.

For the last time point of the rotation we determine the orientation ϕ_N of the ring from the kymograph as described above. For earlier time points, however we make use of the average DV velocity v_{DV} as measured by PIV. From this, we compute an angular velocity $\Omega = v_{\mathrm{DV}}/R$ where R is the average radius of the outline of the cortex determined from the cross-section in the DV-LR plane. Then, the orientation ϕ_j at time point j is computed as

$$\phi_j = \phi_N - \sum_{k=j}^{N} \Omega_k \times 2s, \qquad [6]$$

where Ω_k is the angular velocity between time points k and $k+1$. Thereby, we circumvent that the ring is often found outside of the cortical plane for early time points which can make the orientation determined from intensity measurements in the cortical plane unreliable (See Fig. S5B). This method is based on the observation that movements of cortex as well as cytoplasm resemble the rotation of a rigid body, such that the rotational velocity of the cortical plane equals the rotational velocity of the cytokinetic ring independent of the position of the cytokinetic ring. The ring intensity M_{raw} was determined using linear regression, fitting the function

$$f\,(\theta) = f_0 + M_{\mathrm{raw}} \cos\,(2\theta - \phi_j) \qquad [7]$$

to the intensity values of time point j from the cortical kymograph. Also in the absence of a cytokinetic ring, NMY-2:GFP intensities are never uniform in the cortex, which often yields negative vales for M_{raw} for early time points (Fig S7A). . These inhomogeneities and the resulting M_{raw} at early time points differ in position and magnitude between embryos. However, we find that the trajectories of M_{raw} collapse onto an exponential curve $\exp[\lambda t]$ with a common growth rate $\lambda = 1/(13\mathrm{s})$ for early time points, when allowing for a constant embryo-specific offset (S7E). The offset M_0 is determined by fitting the function

$$f(t) = M_0 + A\exp[t/13s] \tag{8}$$

to M_{raw} for each embryo using linear regression. With this we define the ring intensity

$$M = M_{\mathrm{raw}} - M_0 \tag{9}$$

which we use in Fig. 3B. Since the ring intensity follows an exponential growth, our coarse-grained model predicts that the ring angle ϕ can be written as

$$\phi(t) = \arctan[\tan\phi(0)\exp[-\alpha/\lambda(M - M(0))]] \tag{10}$$

if the effective ring tension driving alignment is proportional to M (See. Eq. 84). Here α is a constant that depends on the geometry and the effective viscosity of the embryo. We consider a common value of α for all embryos. With this, the model predicts that $\phi - \phi_0$ is a common function (Eq. 10) of $M - M(\phi(t) = \phi_0) = M_{\mathrm{raw}} - M_{\mathrm{raw}}(\phi(t) = \phi_0)$. Here ϕ_0 is an arbitrary reference angle. In Fig. S7G-H, we use $\phi_0 = 30°$ and define $M_{\mathrm{raw},0} = M_{\mathrm{raw}}(\phi(t) = \phi_0)$. We find that the trajectories do indeed collapse onto a common function given by 10. α is determined by fitting a linear curve to $\log\tan\phi(t)$.

To measure cytoplasmic flow in the AB cell in the DV-LR plane, Particle Image Velocimetry (PIV) was performed on the lifeact-mKate2 signal in the time window spanning the AB rotation. First, a mask segmenting the cytoplasm and excluding the cortical signal lifeact-mKate2 signal was generated manually. Subsequently, 3-step PIV(5) was performed with final box size of 22x22 pixels (step-size of 12 pixels, pixel size 0.1058 μm), on a grid with 11 pixel spacing. Cytoplasmic flow vectors were averaged over time to generate a mean cytoplasmic flow field. For visualization the DV component (y-component) of the mean flow vectors was color-coded.

Image pre-processing and analysis of mouse recordings. Mouse zygote recordings were first subdivided into three categories based on the inner diameter of the glass capillary: 1) non-compressed, with an inner diameter of 90 μm or larger, 2) mildly compressed with an inner diameter between 80 and 90 μm, 3) strongly compressed, with an inner diameter of 80 μm or smaller. Inner capillary diameters were manually measured in Fiji. For analysis, we used a time window starting from anaphase onset (as determined from the DIC midplane by the start of sister chromatid segregation) up until closure of the cytokinetic ring. From each time point the midplane images were extracted such that the cytoplasmic movements can be quantified. Movies that had the anaphase spindle aligned orthogonal to the imaging plane (i.e., along the imaging direction) were excluded from the anaysis, as is was not possible to extract the spindle orientation because of the limited axial resolution along z in DIC microscopy. For the remainder, a mask was manually generated that segments the contour of the zona pellucida. Cytoplasmic movements were analyzed within this masked region using a 3-step PIV(5), with a final box size of 60x60 pixels (step size of 20 pixels, pixel size of 0.173 μm), on a grid with 30 pixel spacing.

In order to track the approximate position of the spindle poles without a spindle marker, we first manually identified the position of both spindle poles at anaphase onset, during which the positions of the spindle poles can be determined unambiguously. Subsequently, the cytoplasmic velocities at the spindle pole coordinates were inferred by linearly interpolating the local flow field (obtained from the PIV analysis) using the interp2 function in matlab. For each spindle pole, the interpolated velocity vector was used to update the spindle pole coordinate in the following time point. This operation was then iterated over time points, thereby obtaining the time series of approximate spindle pole coordinates. To validate this method, we visually inspected the tracked spindle pole positions. We found that, at the end of cytokinesis, the spindle poles were in direct contact with the daughter cell nuclei, facing the nearest cell cortex (Movie 11-14). This is as expected for the spindle poles, demonstrating the validity of our approach. After inferring the spindle pole coordinates in each time point, the pole-to-pole distance (in μm) and the angle (in degrees) with the capillary long axis were extracted and plotted in polar coordinates.

In order to measure the rotation of the cytoplasm, the curl of the cytoplasmic flow field (obtained by PIV) was calculated using the MATLAB function `curl`. The average curl of a flow field on flat surface $\mathcal{S}$ yields an angular velocity. According to Stokes theorem, this angular velocity corresponds to the flux along the bounding contour $\partial\mathcal{S}$:

$$\Omega = \frac{\int_{\mathcal{S}} \mathrm{d}S\,\mathbf{z}\cdot(\nabla\times\mathbf{v})}{2\int_{\mathcal{S}}\mathrm{d}S} = \frac{\int_{\partial\mathcal{S}}\mathrm{d}\mathbf{l}\cdot\mathbf{v}}{\int_{\partial\mathcal{S}}\mathrm{d}\mathbf{l}\cdot r\boldsymbol{\theta}} \tag{11}$$

Thus, the average curl of the cytoplasm yields two times the angular velocity $\Omega_{\mathrm{cytoplasm}}$ of the cytoplasm at the interface to the cell cortex. Comparison of this angular velocity with the angular velocity of the mitotic spindle revealed very good agreement (Fig. S8E), which is consistent with the notion that the cytoplasm couples cortical rotation to spindle rotation due to viscous drag.

Supplementary Note on Mouse Zygote Division Asymmetry

We noted that the first cell division in mouse zygotes inside glass capillaries was asymmetric in 8 out of 17 embryos (Fig. S9A-B), which is not expected for the first cell division. This cell division asymmetry did not correlate with the strength of compression (Fig. S9C) and it was not observed in control embryos cultured outside glass capillaries (Fig S9B). Therefore, we argue that asymmetric cell division can occasionally be triggered by the procedure of placing zygotes inside glass capillaries, and/or by performing time lapse imaging. To determine whether overall development can progress normally inside glass capillaries, we followed embryo development over the course of 5 days in a separate experiment. 8 out of 8 embryos inside glass capillaries developed into the morula stage, and 6 out of 8 developed into hatched blastocysts (Fig S9D).

We speculate that the abnormal cell division asymmetry observed in a subset of zygotes may have several underlying causes: 1) As the embryos are put inside a glass capillary, they underwent mechanical deformations during sample preparation. 2) The embryos are put into the capillaries more than 12 hrs prior to the first cytokinesis, and the imaging was started more than 5 hrs prior to cytokinesis. Even though the capillaries (approximately 2 cm in length) were open on both sides and were immersed in a bath of KSOM culture media, the media inside the capillary may locally have deteriorated in this time window due to limited exchange with the surroundings. 3) Although time lapse imaging was done using a stage incubator with humidity, temperature and CO2 control, the conditions may not have been as stable as in a conventional incubator. All these factors may have contributed to the deviation from normal cell division symmetry.

Supplementary Notes on Physical Theory

Hydrodynamic model of the *C. elegans* embryo during AB anaphase.

We want to understand how a rotation of the *C. elegans* embryo arises from mechanical interactions within the cortex and between cortex and spindle. To this end, we model the surface of the embryo as a two-dimensional continuous material with spherical topology. We consider the shape of this surface to be defined by the shape of the egg-shell, as cytoplasmic pressure pushes the surface of the embryo against the rigid egg-shell. Forces within the embryo that are normal to the surface such that they would drive a deformation of this surface are balanced by forces from the egg-shell. In this coarse-grained model, we do not distinguish between cortex and cell membrane and treat the interface between AB and P1 cell as part of the bulk material enclosed by the surface. For simplicity, we will model the surface as a fluid film with homogeneous viscosity. To understand how active forces drive flows in this curved geometry, we will use a covariant formalism as derived in (6).

Force balance in a curved surface. The surface of the embryo corresponds to a two-dimensional closed manifold with spherical topology. It can be parmetrised parametrised as $\mathbf{X}(s^1, s^2)$, which defines a covariant basis as

$$\mathbf{e}_i = \partial_i \mathbf{X}, \quad [12]$$

where $i \in \{1, 2\}$, and an outward pointing normal vector

$$\mathbf{n} = \mathbf{e}_1 \times \mathbf{e}_2 / |\mathbf{e}_1 \times \mathbf{e}_2|. \quad [13]$$

In a later section, an explicit parametrisation for an axisymmetric surface is given with the basis vectors illustrated in Fig. S10a.

The covariant basic defines a metric tensor as

$$g_{ij} = \mathbf{e}_i \cdot \mathbf{e}_j \quad [14]$$

The inverse g^{ij} defines the contravariant basis

$$\mathbf{e}^i = g^{ij} \mathbf{e}_j \quad [15]$$

With this, any vector field $\mathbf{f}$ on the surface, e.g. a force field, can be written as

$$\mathbf{f} = f_n \mathbf{n} + f^i \mathbf{e}_i, \quad [16]$$

where here and in the following we use Einstein sum convention. f_n denotes the component of $\mathbf{f}$ that is normal to the surface, whereas $f^i = \mathbf{e}^i \cdot \mathbf{f}$ denote tangential components.

In a hydrodynamic model, conservation of momentum implies that momentum can only be transported in terms of a flux. The flux of momentum within the surface in direction i is given by $-\mathbf{t}^\mathbf{i}$, where

$$\mathbf{t}^i = t^{ij} \mathbf{e}_j + t^i_n \mathbf{n} \quad [17]$$

is the stress (or tension) tensor of the surface material. Then, momentum conservation yields the force balance equation

$$\nabla_i \mathbf{t}^i = -\mathbf{f}_{\text{in}} - \mathbf{f}_{\text{out}} - \rho \mathbf{a}, \quad [18]$$

Here, ∇_i denotes the covariant derivative. $\mathbf{f}_{\text{in}} = f^i_{\text{in}} \mathbf{e}_i + f_{\text{in},n} \mathbf{n}$ is the density of the force the inside of the embryo, in particular spindle and cytoplasm, exert on the surface, and $\mathbf{f}_{\text{out}} = f^i_{\text{out}} \mathbf{e}_i + f_{\text{out},n} \mathbf{n}$ is the force density the surrounding material, in particular the egg-shell, exerts on the surface. $\rho \mathbf{a}$ corresponds to an inertia force resulting from a local acceleration $\mathbf{a}$ and a

mass density ρ. In the following, we will neglect such inertial terms as we will consider a fluid film at low Reynolds number. Expressed in terms of normal and tangential components, the force balance equation then becomes

$$\nabla_j t^{ji} + C^i{}_j t^j_n = - f^i_{\text{out}} - f^i_{\text{in}} \tag{19}$$

$$\nabla_i t^i_n - C_{ij} t^{ij} = - f_{\text{out},n} - f_{\text{in},n}, \tag{20}$$

where $C_{ij} = -\mathbf{n} \cdot \partial_i \partial_j \mathbf{X}$ is the curvature tensor (corresponding to the second fundamental form). We observe, that for non-vanishing curvature of the embryo surface, in-pane cortical tension t_{ij} results in a force density $C_{ij}t^{ij}$ normal to the embryo surface.

Constitutive equations. Here, we consider the shape of the surface to be fixed by the egg-shell, i.e. the normal velocity v_n of the fluid film vanishes. We understand this as a result of the cytoplasmic pressure that pushes the surface of the embryo against the rigid egg-shell (see the following section for a more detailed discussion). Then, Eq. 20 provides a definition for the normal force $f_{\text{out},n}$ the egg-shell exerts on the embryo surface. For the tangential component of the force from the egg-shell, we use a simple friction force, i.e.

$$f^{\text{out}}_i = -\gamma v_i, \tag{21}$$

where γ is a friction coefficient which does not depend on space and time and v_i is the tangential flow field of the embryo surface. We model this surface as a compressible fluid film with active contractility χ such that the deviatoric tangential stress tensor reads

$$t^{ij}_{\text{d}} = \eta \left(2\tilde{v}^{ij} + \nu g^{ij} \nabla_k v^k\right) + \chi g^{ij}. \tag{22}$$

Here, η is the shear viscosity and $\nu\eta$ is the bulk viscosity with ν being a dimensionless number. $\tilde{v}^{ij}$ is the shear rate tensor defined as

$$\tilde{v}^{ij} = \frac{1}{2}\left(\nabla^i v^j + \nabla^j v^i - g^{ij} \nabla_k v^k\right). \tag{23}$$

This corresponds to the minimal model of the actomyosin cortex used in (7). The active isotropic stress χ corresponds to a density of force dipoles within the surface resulting from the activity of motor molecules, in particular Myosin. For a contractile cortex, $\chi > 0$. In general, the stress tensor contains also equilibrium contributions $\mathbf{t}^{\mathbf{i}}_{\mathbf{e}}$ resulting from the free energy of the fluid film in the absence of activity. The tangential force resulting from the equilibrium stress is given by a Gibbs-Duhem relation (6) and reads

$$\mathbf{e}_j \cdot \nabla_i \mathbf{t}^i_e = -\sum_I c^I \partial_j \mu^I, \tag{24}$$

where c^I and μ^I are concentration and chemical potential of chemical species I respectively. This corresponds to a pressure resulting from concentration gradients. Here, we consider a regime where exchange with the cytoplasm limits differences in chemical potential such that the resulting force density (given in Eq. 24) is small compared to the force density $\nabla_i t_d{}^i{}_j$ resulting from viscosity and active stress. Hence, the equilibrium stress does not contribute to the tangential force balance equation which defines the flow field. This allows us to omit the equilibrium stress in the following, where we discuss the flow field of a non-deforming surface. For simplicity we also do not consider deviatoric contributions to the bending moment that would give rise to a normal stress t^i_{n}. Then, we have

$$t^{ij} = t^{ij}_{\text{d}}, \quad t^i_{\text{n}} = 0. \tag{25}$$

The tangential force balance equation (Eq. 19) then reads

$$\gamma v_i = F^{\text{visc}}_i + F^{\text{res}}_i, \tag{26}$$

with

$$F^{\text{visc}}_i := \eta \left((\nu+1)\partial_i(\nabla_k v^k) + \epsilon^j{}_i \partial_j(\epsilon^{kl} \nabla_k v_l) + 2\kappa v^i\right) \tag{27}$$

$$F^{\text{res}}_i := \partial_i \chi + f^{\text{in}}_i, \tag{28}$$

where ϵ_{ij} is the antisymmetric Levi-Cevita tensor and $\kappa = \det C_i{}^j$ is the Gaussian curvature. We have used here that the commutator of the covariant derivative on a curved surface can be written as

$$[\nabla_i, \nabla_j] v^k := (\nabla_i \nabla_j - \nabla_j \nabla_i) v^k = \kappa(\delta^k_i g_{jl} - g_{il}\delta^k_j) v^l. \tag{29}$$

We do not specify $\mathbf{f}_{\text{in}}$ at this point, except that it must be due to interactions within the embryo. This means that it can be written in terms of the three-dimensional stress tensor $\sigma_{\alpha\beta}$ of the bulk of the embryo as

$$f^{\text{in}}_\alpha = - \sum_{\beta \in \{x,y,z\}} \sigma_{\alpha\beta} n_\beta, \quad \alpha \in \{x, y, z\}. \tag{30}$$

Here, n_β are the cartesian components of the outward pointing normal vector of the surface. We do not consider external forces acting on the bulk of the embryo like gravitation, implying

$$\sum_{\beta\in\{x,y,z\}} \partial_\beta \sigma_{\alpha\beta} = 0. \tag{31}$$

Hence, the net force the embryo exerts on the surface has to vanish, i.e.

$$\int_{\mathcal{S}} \mathrm{d}S \mathbf{f}_{\mathrm{in}} = -\int_{\mathcal{V}} \mathrm{d}V \partial_\beta \sigma_{\alpha\beta} = 0, \tag{32}$$

where $\mathcal{V}$ is the bulk volume of the embryo. We also do not consider external torques, e.g. from a magnetic field. Therefore, angular momentum conservation implies that $\sigma_{\alpha\beta}$ is symmetric and that the net torque the embryo exerts on the surface has to vanish, i.e.

$$\int_{\mathcal{S}} \mathrm{d}S \mathbf{X} \times \mathbf{f}_{\mathrm{in}} = 0. \tag{33}$$

We also do not consider external forces acting on the surface. This implies that the net force and torque the egg-shell exerts on the surface of the embryo has to vanish:

$$\int_{\mathcal{S}} \mathrm{d}S \mathbf{f}_{\mathrm{out}} = 0 = \int_{\mathcal{S}} \mathrm{d}S \mathbf{X} \times \mathbf{f}_{\mathrm{out}} \tag{34}$$

This turns out to be crucial for understanding the compression-triggered rotation.

Geometry of an almost axisymmetric surface . In uncompressed conditions, the shape of the egg-shell and, hence, the embryo is almost axi-symmetric. Hence, the shape of the embryo surface can be written as

$$\mathbf{X}(\theta, s) = (\rho(s)\cos\theta,\ \rho(s)\sin\theta,\ z(s))^T = \rho(s)\boldsymbol{\rho} + z(s)\mathbf{z}. \tag{35}$$

Here, s is the arc-length corresponding to the AP direction on the cortex and θ is the azimuthal angle. ρ is the distance from the z axis connecting the AP poles. In the arc-length parametrisation ρ and z define an angle $\psi(s)$ via $(\cos\psi, \sin\psi) = (\rho'(s), z'(s))$ (see Fig. S10a). With this, the local basis vectors are given by

$$\mathbf{e}_\theta = \rho\boldsymbol{\theta}, \quad \mathbf{e}_s = \cos\psi\boldsymbol{\rho} + \sin\psi\mathbf{z}, \tag{36}$$

where $\boldsymbol{\theta}, \boldsymbol{\rho}, \mathbf{z}$ are normalized vectors. The curvature tensor is given by

$$C_s{}^s = \psi'(s), \quad C_\theta{}^\theta = \frac{\sin\psi}{\rho}. \tag{37}$$

For small deviations from axisymmetry, the shape can be written as

$$\mathbf{X}' = \mathbf{X} + \delta X_n \mathbf{n}_0, \tag{38}$$

where $\mathbf{n}$ is the outward pointing normal vector given by

$$\mathbf{n} = \sin\psi\boldsymbol{\rho} - \cos\psi\mathbf{z}. \tag{39}$$

Upon such a deformation, the basis vectors change in first order of the deformation as

$$\delta\mathbf{e}^i = -\,\delta X_n C^i{}_j \mathbf{e}^j + g^{ij}(\partial_j \delta X_n)\mathbf{n}, \tag{40}$$

$$\delta\mathbf{n} = -\,(\partial_i \delta X_{\mathrm{n}})\mathbf{e}^i = -\rho(\partial_\theta \delta X_n)\boldsymbol{\theta} - (\partial_s \delta X_n)(\cos\psi\boldsymbol{\rho} + \sin\psi\mathbf{z}). \tag{41}$$

Note on cytokinetic furrow ingression in a confined cell.
From the normal force balance Eq. 20, we read off that tension in the curved actomyosin cortex yields forces perpendicular to the cell surface. In an unconfined cell, these normal forces balance a difference in pressure between the cytoplasm and the fluid surrounding the cell:

$$\nabla_i t^i_{\mathrm{n}} - C_{ij} t^{ij} = P_{\mathrm{out}} - P_{\mathrm{in}}, \tag{42}$$

where P_{out} and P_{in} correspond to ambient and cytoplasmic pressure, respectively. Bending rigidity of the cell surface yields an additional normal force density $\nabla_i t^i_{\mathrm{n}}$ (6, 7). (For simplicity, we neglect here any shear stresses from cytoplasm or the surrounding fluid.) When the cytokinetic ring forms, myosin accumulation yields an active tension t^{ij}_{act} that drives a deformation corresponding to an ingressing furrow whose shape can be calculated from Eq. 42 (see (4) for an explicit calculation).

In the *C. elegans* AB cell, most of the surface is in contact with the rigid egg-shell that confines the embryo. We understand this as a result of the cytoplasmic pressure, in particular the osmotic pressure, that presses the embryo surface against the

egg-shell. In this regime, the egg-shell fixes the shape of the embryo by exerting inward forces $f_{\mathrm{out,n}}$ on the embryo surface balancing the residual outward force per area that results from cytoplasmic pressure and cortical tension:

$$-f_{\mathrm{out,n}} = \nabla_i t^i_{\mathrm{n}} - C_{ij} t^{ij} + P_{\mathrm{in}} \quad [43]$$

In this regime, changes in cortical tension do not yield a deformation of the cell but a change in the egg-shell force density $f_{\mathrm{out,n}}$. In particular, inward forces generated by the cytokinetic ring are balanced by a reduction of the inward forces $f_{\mathrm{out,n}}$ the egg-shell exerts on the embryo surface. In this sense, inward forces generated by the ring yield outward forces from the egg-shell as mentioned in the main text.

About a minute after Myosin has started to accumulate in the cytokinetic ring, the cytokinetic ring detaches from the egg-shell forming the cytokinetic cleavage furrow that will ingress further and separate the future daughter cells (4). Upon detachment, any outward force that the cell exerts on its surrounding at the cytokinetic ring is no longer balanced by the egg-shell but the perivitelline fluid (see Eq. 42). This implies a critical force density for ring ingression:

$$f_{\mathrm{ingression}} := P_{\mathrm{in}} - P_{\mathrm{out}} \quad [44]$$

given by the pressure difference between cytoplasm and perivitelline fluid. The ring ingresses as soon as the active tension t^{ij}_{act} in the cytokinetic yields a normal force density that is greater than this critical force density, when keeping the surface fixed, i.e.

$$C_{ij}(t^{ij}_{\mathrm{act}} + t^{ij}_{\mathrm{visc}}) - \nabla_i t^i_{\mathrm{n}} > f_{\mathrm{ingression}}, \quad [45]$$

when imposing $v_{\mathrm{n}} = 0$. Note that on the left-hand side also the viscous stress t^{ij}_{visc} and the normal stress t^i_{n} contribute. For a given rate of myosin accumulation in the cytokinetic ring, the critical force for ingression yields a time-lag between ring formation and ingression. Such a time-lag has been reported previously in the *C. elegans* embryo (4). Also in the mouse zygotes that we imaged under strong compression, such a time-lag is evident. This can be seen from the cytoplasmic flow 10min. after anaphase onset in the mouse zygote in Fig. S8C. At this time-point, no ingression of the cytokinetic furrow is observed. At the same time, we observe considerable cytoplasmic flows indicating that the cytokinetic ring that drives convergent flows in cortex and cytoplasm has already formed. The uncompressed zygote in Fig. S8A, in contrast, has already started to deform at a comparable time point ($t = 10$min.), even though the cytoplasmic flows are still weaker than in the strongly compressed zygote.

Rotation triggered by compression.

Compression triggers rotation as a consequence of torque balance. Experimentally, we find that the compression of *C. elegans* embryo, induces a whole-embryo rotation around the AP axis during anaphase of the AB cell, resembling the rotation of a rigid body. In the absence of compression, no such rotation is observed. This can be understood as a consequence of torque balance (Eq. 34). To this end, let us write the velocity field as

$$\mathbf{v} = \Omega\rho\boldsymbol{\theta} + \mathbf{v}_{\mathrm{res}}, \quad [46]$$

where

$$\Omega := \frac{1}{\Theta_{zz}} \mathbf{z} \cdot \int_{\mathcal{S}} \mathrm{d}S \mathbf{X} \times \mathbf{v}, \quad \Theta_{zz} = \int_{\mathcal{S}} \mathrm{d}S \rho^2, \quad [47]$$

$$\mathbf{v}_{\mathrm{res}} := \mathbf{v} - \Omega\rho\boldsymbol{\theta}. \quad [48]$$

Ω corresponds to the component of the flow field that defines a (rigid body) rotation around the z axis. The friction force acting against this rotation yields a torque given by

$$T_{\mathrm{fric}} = -\gamma \mathbf{z} \cdot \int_{\mathcal{S}} \mathrm{d}S \mathbf{X} \times \mathbf{v} = -\gamma \Theta_{zz} \Omega. \quad [49]$$

However angular momentum dictates that the net torque the egg-shell exerts on the embryo surface has to be zero:

$$0 = T_{\mathrm{out}} = \mathbf{z} \cdot \int \mathrm{d}S(\mathbf{X} \times \mathbf{f}_{\mathrm{out}}) = T_{\mathrm{fric}} + T_{\mathrm{n}}. \quad [50]$$

The torque due to normal forces

$$T_{\mathrm{n}} = \mathbf{z} \cdot \int_{\mathcal{S}} \mathrm{d}S \mathbf{X} \times (f_{\mathrm{out,n}} \mathbf{n}) \quad [51]$$

vanishes for an axisymmetric surface, because

$$\mathbf{z} \cdot (\mathbf{X} \times \mathbf{n}) = 0. \quad [52]$$

This can be understood from a cross-section perpendicular to $\mathbf{z}$ which is a circle for an axisymmetric surface (Fig. S10). Hence, the torque from friction force $T_{\mathrm{fric}} = -T_{\mathrm{n}}$ has to vanish. Thus, the rigid body rotation Ω of an axisymmetric surface has to vanish in the absence of friction gradients or external torques.

Upon compression, a rapid rotation of the embryo is observed during AB anaphase. To understand the effect of compression, we consider a small deformation $\delta X_n << R$ of the embryo surface relative to some axisymmetric reference shape with R being the average diameter of the embryo in the LR-DV plane. With this we show in the following that the compression-triggered rotation can be understood from the balance of torques acting on the embryo surface. In the next section, we explicitly calculate how the flow field on the surface of a spherical cell changes upon a small deformation and find that the compression-triggered rotation dominates in the regime of vanishing friction.

On the slightly deformed axisymmetric surface, we write the change in the flow field with respect to a reference flow field $\mathbf{v}_0$ on the axisymmetric surface as

$$\delta\mathbf{v} = \delta\mathbf{v}_{\text{res}} + \delta\Omega\rho\boldsymbol{\theta}, \quad \delta\Omega := \frac{1}{\Theta_{zz}}\int_{\mathcal{S}_0} \mathrm{d}S\rho\boldsymbol{\theta}\cdot\delta\mathbf{v}. \qquad [53]$$

With this we can write the torque balance equation (Eq. 34) in linear order of δX_n as

$$\delta T_{\text{n}} = -\delta T_{\text{fric,geo}} + \gamma\Theta_{zz}\delta\Omega, \qquad [54]$$

where

$$\begin{aligned}\delta T_{\text{n}} =& \mathbf{z}\cdot\int_{\mathcal{S}} \mathrm{d}S\mathbf{X}\times(f_{n,0}^{\text{out}}\delta\mathbf{n}) = -\int_{\mathcal{S}} \mathrm{d}S f_{n,0}^{\text{out}}\partial_\theta\delta X_n \\ =& \int_{\mathcal{S}} \mathrm{d}S(f_{n,0}^{\text{in}} - C_{ij}t_0^{ij})\partial_\theta\delta X_n \qquad [55]\\ \delta T_{\text{fric,geo}} =& -\mathbf{z}\cdot\gamma\int_{\mathcal{S}} \mathrm{d}S\left(\delta\mathbf{X}\times\mathbf{v}_0 + \frac{\delta\sqrt{g}}{\sqrt{g}}\mathbf{X}\times\mathbf{v}_0\right) \\ =& -\gamma\int_{\mathcal{S}} \mathrm{d}S(\rho\psi' + 2\sin\psi)\delta X_n\boldsymbol{\theta}\cdot\mathbf{v}_0. \qquad [56]\end{aligned}$$

Here, δT_{n} is a torque that results from egg-shell normal forces balancing embryo-internal stresses in the presence of a non-axisymmetric deformation δX_n, as the azimuthal gradient of the deformation $\partial_\theta\delta X_n$ yields a normal vector that is no longer parallel to $\mathbf{X}$ in the LR-DV plane (see Eq. 41 and Fig. S10c). This torque results from an azimuthal misalignment of the pattern of normal forces $f_{n,0}^{\text{out}}$ and the geometry defined by δX_n. This can be seen by expanding $f_{n,0}^{\text{out}}$ and δX_n as a Fourier series

$$f_{n,0}^{\text{out}}(\theta, s) = \sum_{k\in\mathbb{N}} f_k(s)\cos[k(\theta - \phi_k^f(s))] \qquad [57]$$

$$\delta X_n(\theta, s) = \sum_{k\in\mathbb{N}} d_k(s)\cos[k(\theta - \phi_k^X(s))], \qquad [58]$$

yielding

$$\delta T_{\text{n}} = \pi\sum_{k\in\mathbb{N}}\int_0^L \mathrm{d}s\rho f_k d_k \sin[k(\phi_k^f - \phi_k^X)], \qquad [59]$$

where we observe that δT_{n} arises from an azimuthal misalignment $\phi_k^f - \phi_k^X$ of the pattern of normal forces and the non-axisymmetric geometry.

This torque results in a rotation driving an alignment of force pattern and geometry. For simplicity and motivated by the experimentally observed speed of the compression-triggered rotation, we consider here a regime of small friction with the egg-shell, i.e. a large hydrodynamic length $\eta/\gamma >> R^2$. Then, the friction forces $\gamma\mathbf{v}_0$ resulting from the cortical flow towards the cytokinetic ring are small compared to the normal forces $f_{n,0}^{\text{in}} - C_{ij}t_0^{ij}$ that drive ingression of the cyokinetic ring and expansion of the cell poles. In this regime, misalignment of normal forces and geometry result in a torque T_n that is large compared to the torque from friction forces $T_{\text{fric,geo}}$, such that Eq. 54 simplifies to

$$\delta\Omega = \frac{1}{\gamma\Theta_{zz}}\delta T_{\text{n}} = \frac{\pi}{\gamma\Theta_{zz}}\sum_{k\in\mathbb{N}}\int_0^L \mathrm{d}s\rho f_k d_k \sin[k(\phi_k^f - \phi_k^X)]. \qquad [60]$$

This means that an misalignment $\phi_k^f - \phi_k^X$ between normal forces and geometry triggers a rotation, whenever friction forces with the egg-shell are small compared to the normal forces triggering the rotation. In the regime of small friction we are considering here, the rotation will be fast, i.e. $\delta\Omega\rho >> \langle|\mathbf{v}_0\delta X_n/\rho|\rangle$. Importantly, a rigid body rotation does not contribute to the normal force $C_{ij}t_0^{ij}$ resulting from viscous forces in the axisymmetric surface. Hence, Eq. 60 remains valid in first order of δX_n up to $\delta\Omega\rho$ being of order $\mathbf{v}_0$ at which point viscous forces will limit the rotation. In the AB cell, the compression-independent flow speed prior to chiral flows is about 7μm/min. (8). We find that the speed of the compression-triggered rotation is about $0.5 - 2$deg./s (Fig. 3) for aspect ratios $AR < 0.95$, corresponding to a cortical flow velocity of $10 - 30\mu$m/min. (see also Fig. S7B for direct measurements of cortical flow). This suggests that the linear approximation is valid up to $AR \sim 0.95$,

corresponding to $\delta X_n \sim \delta\rho \sim 0.3\mu$m, where $\delta\Omega\rho_0 \sim |v_0| = 7\mu$m/min. and hence $\gamma \sim (\eta/\rho^2)(\delta\rho/\rho_0)$. This yields an estimate for the hydrodynamic length,

$$l_h = \eta/\gamma \sim 100\mu\text{m}, \tag{61}$$

which is consistent with the observation that the entire embryo of length $\sim 50\mu$m rotates like a rigid body.

In the absence of external torques and for small deformations δX_n, the bulk of the embryo will move along with this rigid-body-like rotation of the embryo surface. As the pattern defining the normal forces rotates with the embryo, the rotation (Eq. 60) results in an alignment

$$\partial_t \phi_k^f \sim \delta\Omega \tag{62}$$

towards

$$\phi_k^f - \phi_k^X = 0 + 2nk\pi, \quad n \in \mathbb{N}, \tag{63}$$

for $d_k f_k < 0$. In other words, the deformation-triggered rotation azimuthally aligns patches that pull (push) on the egg-shell such that $f_\text{n}^\text{out} > 0$ ($f_\text{n}^\text{out} < 0$) with points in the geometry that are deformed inward (outward) relative to the axisymmetric surface, i.e. $\delta X_n < 0$ ($\delta X_n > 0$).

A compression defining a long and a short axis in the LR-DV plane corresponds to a deformation

$$\delta X_n \approx d_2(s)\cos(2(\theta - \phi_2^X)). \tag{64}$$

For $d_2(s) > 0$, ϕ_2^X is the azimuthal angle of the long axis. The compression-triggered rotation aligns this long axis with the axis of the azimuthal pattern of normal forces, i.e. the $k = 2$ component of f_n^out (Eq. 57). For the AB cell dividing in the LR-DV plane with ingressing ring and expanding poles, ϕ_2^f corresponds to the angle of the spindle axis for $f_2 < 0$. Hence, the compression-triggered rotation aligns the spindle axis with the long axis of the cell in the LR-DV plane.

This suggests that Hertwig's rule, i.e. cells dividing along their long axis, is a robust consequence of torque balance, whenever the surface of the cell is free to rotate and the surrounding resists ingression of the ring (or expansion of the cell poles). Torque balance also provides an explanation for the spindle orientation in embryos with inhibited actomyosin contractility and enhanced astral pulling forces. In these embryos, we expect the pulling forces at the poles to dominate over ingressing forces at the ring. Thereby, we obtain from torque balance a rotation that aligns the spindle with the short axis in the LR-DV plane. We want to stress that this result only depends on the normal forces exerted on the surface in the absence of the deformation δX_n. Hence it does not require detailed knowledge about the nature of spindle-cortex interactions. It only depends on whether astral microtubules are pushing or pulling at the cortex. Furthermore, we note that this argument is also valid for a scenario where spindle anchors move through the cortex such that the cortex acts as an effectively rigid surface. In this case, spindle-cortex interactions would drive a rotation of the spindle relative to a static cortex and egg-shell. Given the high viscosity of the cortex and the limited number of anchors, such a scenario seems likely.

Deformation-triggered rotation of a spherical cell. In the following, we give explicit results for the change in cortical flow that results from statically deforming a spherical cell. This includes the deformation-triggered rotation.

We parametrize the surface as

$$\mathbf{X}(\theta,\varphi) = (R + \delta R(\theta,\varphi))\mathbf{r}(\theta,\varphi), \tag{65}$$

where

$$\mathbf{r}(\theta,\varphi) = (\cos\phi\sin\theta, \sin\phi\sin\theta, \cos\theta)^T. \tag{66}$$

$\delta R(\theta,\varphi)$ corresponds to a normal deformation of the spherical surface, corresponding to δX_n in the previous section. To compute the flow field we use a Hodge decomposition:

$$v_i = \partial_i A + \epsilon^{ji}\partial_j B, \tag{67}$$

where A is a scalar field corresponding to the irrotational component and B is a pseudoscalar corresponding to the rotational component of the tangential flow field v_i. We expand these (pseudo-)scalar fields as well as the deformation δR and the contractility χ in terms of scalar spherical harmonics Y_{lm}:

$$A(\theta,\varphi) = \sum_{l=1}^{\infty}\sum_{m=-l}^{l} A_{lm}Y_{lm}(\theta,\varphi), \quad B = \sum_{l=1}^{\infty}\sum_{m=-l}^{l} B_{lm}Y_{lm}$$
$$\delta R_{lm} = \sum_{l=0}^{\infty}\sum_{m=-l}^{l} \delta R_{lm}Y_{lm}, \quad \chi_{lm} = \sum_{l=0}^{\infty}\sum_{m=-l}^{l} \chi_{lm}Y_{lm} \tag{68}$$

The flow field for $\delta R_{lm} = 0$ is given by

$$A_{lm}^0 = \frac{\chi_{lm}/\eta}{1/l_h^2 + [(\nu+1)l(l+1) - 2]/R^2}, \quad B_{lm}^0 = 0, \tag{69}$$

where the hydrodynamic length is defined as $l_h = \sqrt{\eta/\gamma}$ For details of the calculation see (7), where also the effect of an enclosed Stokes fluid is considered. For $\delta R \neq 0$, the flow field becomes

$$A_{lm} = A^0_{lm} + \delta A_{lm}, \quad B_{lm} = \delta B_{lm}, \qquad [70]$$

where

$$\delta A_{lm} = \frac{1}{1/l_h^2 + \left[(\nu+1)l(l+1) - 2\right]/R^2} \frac{S_{lm} + (-1)^m \bar{S}_{lm}}{2\eta\sqrt{l(l+1)}} \qquad [71]$$

$$\delta B_{lm} = \frac{-i}{1/l_h^2 + \left[l(l+1) - 2\right]/R^2} \frac{S_{lm} - (-1)^m \bar{S}_{lm}}{2\eta\sqrt{l(l+1)}} \qquad [72]$$

with an effective torque and tension density resulting from the change in viscous forces given by

$$\frac{S_{lm}}{2} = \sum_{l_1,l_2,m_1,m_2} \frac{(-1)^{m+1}\chi_{l_1m_1}\delta R_{l_2m_2}/R}{R^2/l_h^2 + (\nu+1)l_1(l_1+1) - 2} \sqrt{\frac{(2l_1+1)(2l_2+1)(2l+1)}{4\pi}} \begin{pmatrix} l_1 & l_2 & l \\ m_1 & m_2 & -m \end{pmatrix} \left\{ \left[l_2(l_2+1) - 2\right] \sqrt{l_1(l_1+1)} \begin{pmatrix} l_1 & l_2 & l \\ -1 & 0 & 1 \end{pmatrix} - (\nu+1)l_1(l_1+1)\sqrt{l(l+1)} \begin{pmatrix} l_1 & l_2 & l \\ 0 & 0 & 0 \end{pmatrix} \right\}. \qquad [73]$$

Here

$$\begin{pmatrix} l_1 & l_2 & l \\ m_1 & m_2 & m \end{pmatrix} \qquad [74]$$

are Wigner 3j symbols (closely related to the better known Clebsch-Gordan coefficients). They result from the product of two spherical harmonics function projected onto a third spherical harmonic, corresponding to the flow field that results from the couple of deformation and contractility fields. Details of the calculation will be published elsewhere. For small friction, i.e. large hydrodynamic length $l_h > R$, the deformation-dependent flow field resulting from $\delta A_{lm}, \delta B_{lm}$ is dominated by the deformation-triggered rotation given by $\delta B_{1,m}$. Identifying the rotation axis as the z axis such that $\delta\mathbf{\Omega} = \mathbf{z}\delta\Omega$, the rigid body rotation of the deformed sphere is given by

$$\delta\Omega = -\sqrt{\frac{3}{4\pi}}\delta B_{1,0} = i\frac{3}{4\pi\gamma}\sum_{l,m}(-1)^m\chi_{l,m}\delta R_{l,-m}\frac{m[l(l+1)-2)]}{(\nu+1)l(l+1)-2}. \qquad [75]$$

Writing the spherical harmonics coefficients in terms of an azimuthal angle and a magnitude as

$$\delta R_{lm} = |\delta R_{lm}|e^{-im\phi_{R,lm}}, \quad \delta\chi_{lm} = |\delta\chi_{lm}|e^{-im\phi_{\chi,lm}}, \qquad [76]$$

we obtain

$$\delta\Omega = \frac{1}{\gamma}\sum_{l\geq 2, m>0}\frac{3}{2\pi}\frac{m\left[l(l+1)-2\right]}{(1+\nu)l(l+1)-2}|\chi_{l,m}||\delta R_{l,m}|\sin[m(\phi_{\chi,lm} - \phi_{R,lm})] \qquad [77]$$

This equation is equivalent to Eq. 60. We observe again that the rotation results from a misalignment between the heterogeneity of the surface geometry (δR) and the normal force pattern which we can express here directly in terms of the pattern of active tension χ. We observe that $\delta\Omega \to 0$ for $\nu \to \infty$, even if $\nu\eta = \text{const.}$, corresponding to vanishing shear viscosity. For vanishing bulk viscosity $\nu\eta \to 0$, $\delta\Omega$ does not vanish. Hence, the deformation-triggered rotation of a fluid film driven by isotropic active tension results from viscous shear forces.

Dynamics of axis alignment. In the following, we study the dynamics of the cell division axis resulting from the compression-triggered rotation, i.e.

$$\partial_t\phi = \delta\Omega, \qquad [78]$$

where ϕ is the azimuthal angle of the spindle axis of the AB cell and $\delta\Omega$ is the compression-triggered rotation given in Eq. 60. We consider a compression given by

$$\delta X_n \approx d_2(s)\cos(2(\theta - \phi_2^X)), \qquad [79]$$

where ϕ_2^X defines a compression axis that is consistent throughout the embryo, i.e. $\partial_s\phi_2^X = 0$. Also for the normal force pattern we consider a common axis throughout the embryo, which corresponds to the cell division axis, i.e. $\phi_2^f(s) = \phi$. In the following, we choose a coordinate system such that $\phi_2^X = 0$. With this, the dynamics of the spindle axis can be written as

$$\Omega = \partial_t\phi(t) = -W(t)\sin 2\Delta\phi(t), \qquad [80]$$

where W is the magnitude of an effective force that drives alignment and can be expressed in terms of deformation and egg-shell normal force as

$$W(t) = -\frac{\pi}{\gamma \Theta_{zz}} \int_0^L \mathrm{d}s \rho(s) f_2(s,t) d_2(s). \qquad [81]$$

Indeed, we find experimentally that $|\Omega|$ generally increases up to an angle of $\phi = \pm 45^\circ$, i.e. $\sin 2\phi = \pm 1$, with ϕ being the angle of the cytokinetic ring (Fig. S7F). f_2 is the $k = 2$ component of the normal force for an axisymmetric egg-shell as defined in Eq. 57. It is given by the normal force balance equation (Eq. 20) with the flow field obeying the tangential force balance equation (Eq. 19). Hence f_2, is linear in the magnitude of the active tension driving the flow and depends on the viscosity of cortex and cytoplasm. Due to this linearity and rotational symmetry of the surface, f_2 only depends on the $k = 2$ component of the active tension χ, which we interpret as the active tension in the cytokinetic ring $T(t)$ such that we may write

$$W(t) = \alpha T(t) \qquad [82]$$

with α being a constant of proportionality that depends on the viscosities as well as the AP profile (i.e. s dependence) of deformation and active tension. Eq. 77 yields an explicit expression for α for a deformed sphere.

We note that Eq. 80 is not specific to the active fluid model we have discussed in the previous sections. It is a general result for a rotation of an axis, here division axis, driven by misalignment with an external axis, here compression axis in two dimensions. It is solved by

$$\tan \phi(t) = \tan \phi(t_0) \exp\left[- \int_{t_0}^{t} \mathrm{d}t W(t) \right], \qquad [83]$$

i.e. an exponential decay of $\tan \phi$ with a time-dependent decay rate $W(t)$. The physical mechanism driving the alignment defines the time-evolution of this rate. When alignment is driven by actomyosin contractility and contractility scales linearly with Myosin concentration, $W(t)$ is proportional to the $k = 2$ component of Myosin concentration as measured by flourescence microscopy, which we call the relative Myosin concentration M in the cytokinetic ring, see Suppl. Methods for details. Experimentally, we find that the time-evolution of M can be captured by an exponential growth with a common rate $\lambda = 1/(13\mathrm{s})$, consistent with an instability of the cortex triggered by spindle-cortex interactions. Hence, $W(t) \sim T(t) \sim M(t)$ yields

$$\log \tan \phi(t) = \log \tan \phi(t_0) - \alpha \frac{M(t) - M(t_0)}{\lambda}. \qquad [84]$$

Indeed, the experimental trajectories of ϕ collapse onto such a curve (Fig. 4E,S7G,H).

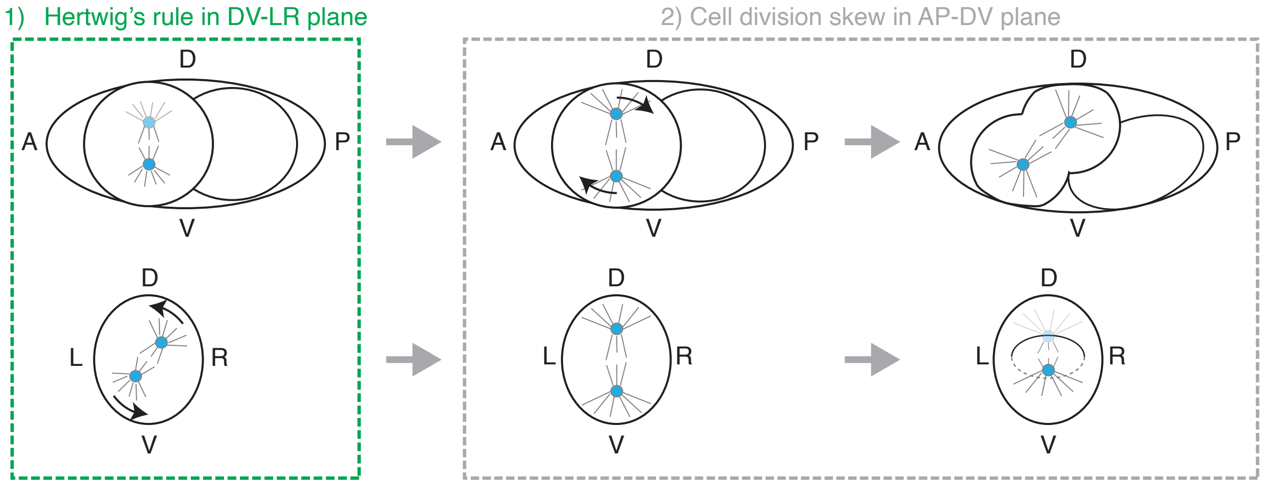

Fig. S1. Hertwig's rule execution in the AB cell
Schematic of the AB cell division viewed in the AP-DV plane (top) and the DV-LR plane (bottom). Hertwig's rule is executed by a spindle rotation during anaphase in the DV-LR plane (left). This occurs before the cell division skew in the AP-DV plane (right).

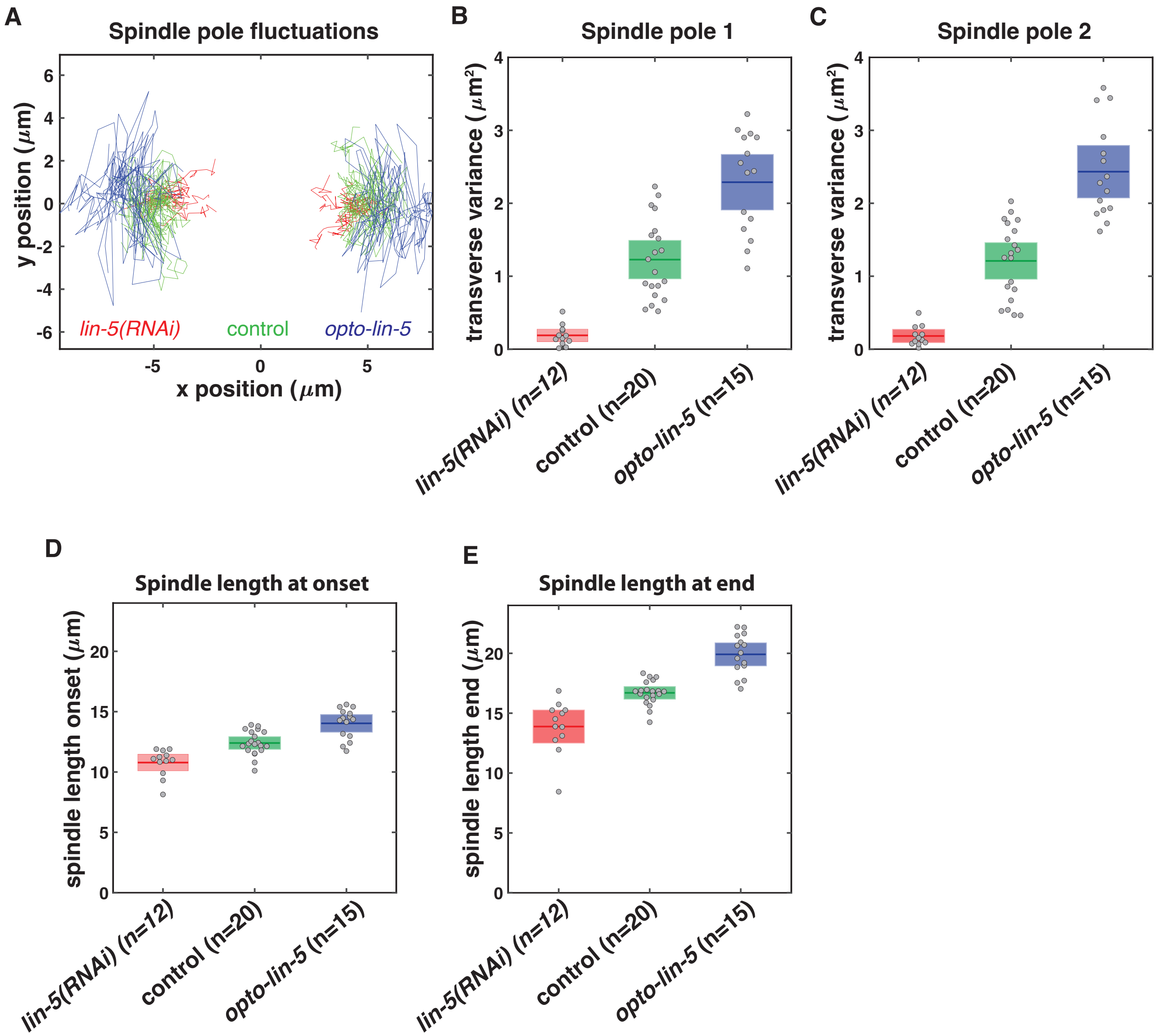

Fig. S2. Modulating cortical LIN-5 levels affects cortical pulling forces on the spindle.
A Spindle pole fluctuations in control (green), *lin-5(RNAi)* (red), and *opto-lin-5* (blue). The spindle poles were tracked in the 15 time points (120 sec) leading up to the onset of rotation, or up to anaphase onset in *lin-5(RNAi)*. Subsequently, for each embryo the spindle pole coordinates were rotated such that the spindle was oriented horizontally, along the x-axis, at the onset of rotation. Figure **A** shows time traces of both spindle pole coordinates in all embryos. **B-C** Variance in y-positions, denoted transverse variance, for each embryo of both spindle poles. Mean over embryos ± 95 percent confidence intervals are indicated. Increasing levels of cortical LIN-5 lead to more movements in the y-direction, i.e. more transverse fluctuations. **D-E** Spindle length (pole-to-pole distance) at onset and at the end of the rotation. The end point is defined by the starting time point of the cell division skew in the AP-DV plane (Fig. S1). The onset is defined as 160 seconds prior to the end point.

A

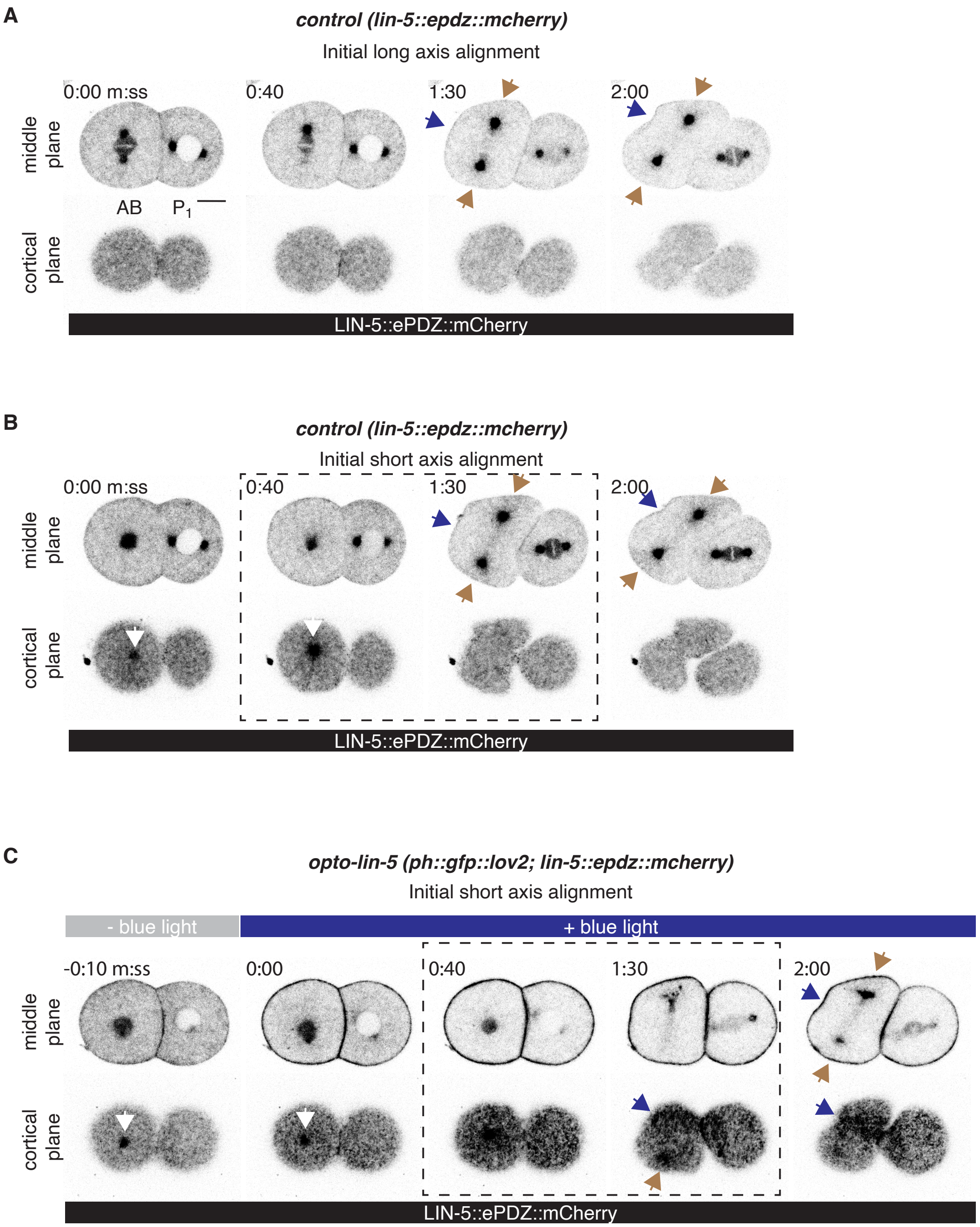

Fig. S3. LIN-5 localizes to the AB cell cortex during mitosis.
A-B Compressed embryos producing endogenously-labeled LIN-5 (*lin-5::epdz::mcherry*) imaged from metaphase (left) to late anaphase (right) in the AP-DV plane. **A** displays an embryo with initial long axis alignment and **B** displays an embryo with initial short axis alignment of the AB spindle. Top panels show the embryo midplane, and bottom panels show the cortical surface. LIN-5 localizes to the cytoplasm and the mitotic spindle, and displays mild, symmetric cortical localization throughout mitosis. During anaphase progression LIN-5 gets mildly enriched at the anterior part of the cytokinetic ring (blue arrow heads) and at the cell poles (brown arrow heads). **C** Endogenously labeled LIN-5 (*lin-5::epdz::mcherry*) in an *opto-lin-5* embryo. First time point shows LIN-5::ePDZ::mCherry prior to global blue-light illumination. Even prior to blue-light illumination there is increased cortical LIN-5::ePDZ::mCherry localization, indicative of dark activity. Upon blue-light illumination there was a uniform global recruitment of LIN-5::ePDZ::mCherry. Upon anaphase progression, LIN-5::ePDZ::mCherry was enriched at the cytokinetic ring (blue arrow heads) and the poles (brown arrow heads). In both control and *opto-lin-5* embryos with initial short axis alignment, we observe a profound localized cortical LIN-5 signal during metaphase (**B-C**, white arrow heads). This may either be the spindle pole which is touching the cortical surface or a localized cortical enrichment. Dashed rectangles mark the timepoints between which the anaphase spindle rotation in the DV-LR plane occurred. Scale bar = 10 μm

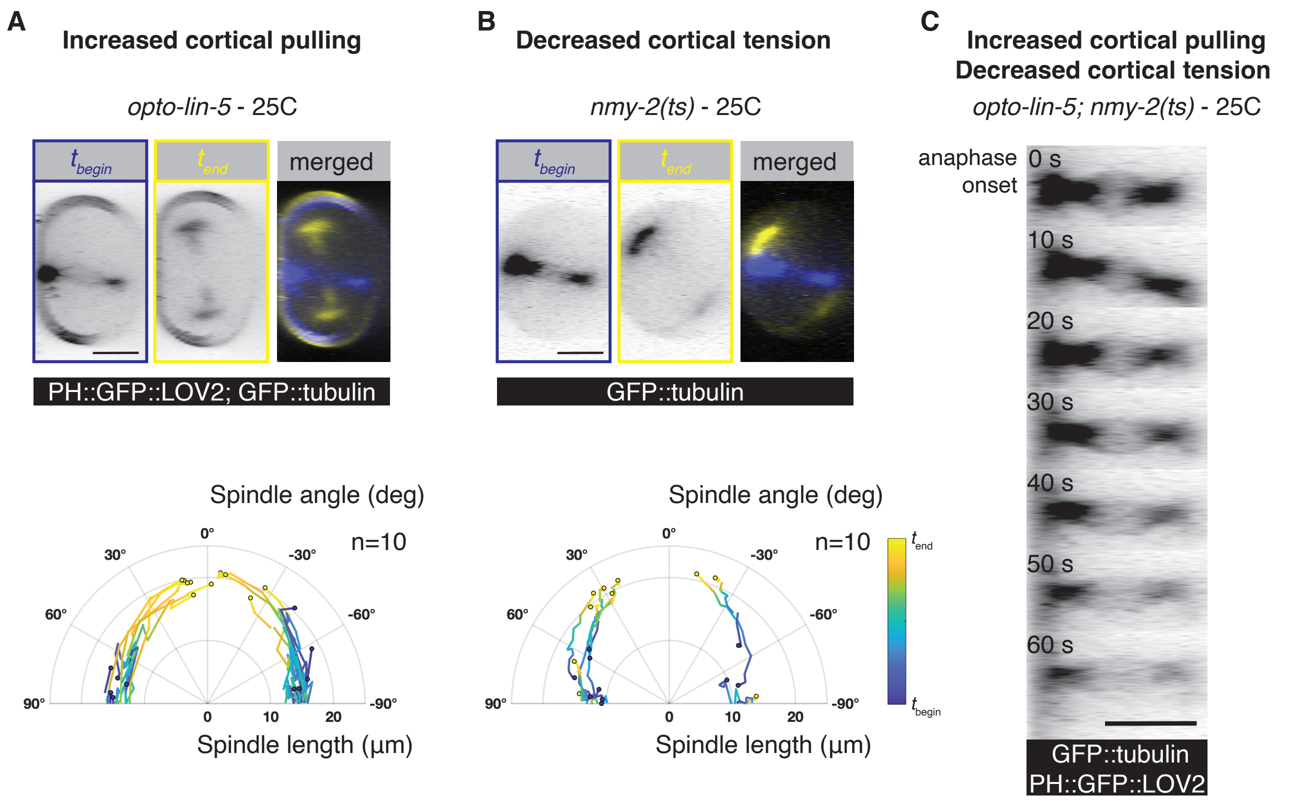

Fig. S4. Controls for the *nmy-2(ts); opto-lin-5* condition in the main text:
Spindle positioning in the DV-LR plane of compressed embryos imaged at 25C upon **A** increased cortical pulling on the spindle - *opto-lin-5*, and **B** reduced cortical tension - *nmy-2(ts)*. Top: still images showing **A** PH::GFP::LOV2; GFP::tubulin and **B** GFP::tubulin at the at the beginning of anaphase (t_{begin}, blue) and at the end (t_{end}, yellow), viewed in the DV-LR plane. Bottom: time evolution of spindle length (pole-to-pole distance) and angle with the long axis plotted in polar coordinates. Traces represent individual embryos. These supplementary figure panels provide the controls for the *opto-lin-5; nmy-2(ts)* experiment (main Fig. 2G), which were done in the absence of the lifeact-mKate2 transgene (unlike main Fig 2E and F) and at 25C (unlike main Fig. 2C). **C** Zoomed still images of the spindle in an *opto-lin-5; nmy-2(ts)* embryo imaged at 25C (same embryo as shown in main Fig 2G) during anaphase progression. Although the spindle in *opto-lin-5; nmy-2(ts)* embryos at 25C does not elongate due limited space along the short axis, anaphase A progresses normally, as evidenced by the shortening of the kinetochore microtubules. Scale bars 10 μm.

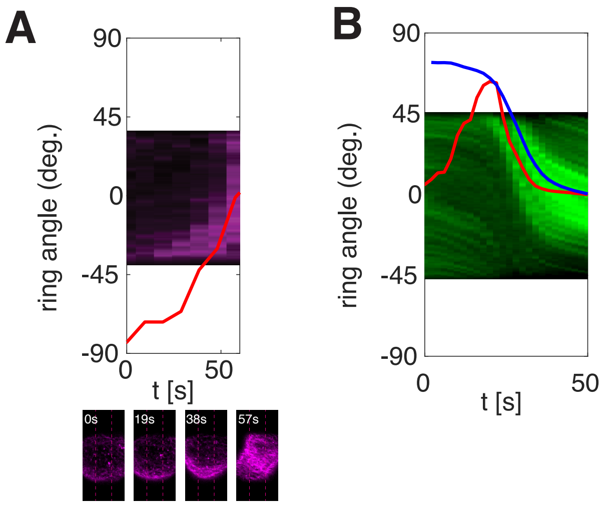

Fig. S5. Inferring ring angle from cortical intensity measurements

A Kymograph of Lifeact::mKate2 intensity from the cortical plane of the embryo in Fig. 3 averaged along AP axis in the window indicated with dashed lines in the micrographs below. Each square corresponds to a pixel along the DV axis. The corresponding azimuthal angle was determined from the cortical outline in the DV-LR plane (see Eq. 3) Using linear regression of the fitting function in Eq. 4, the ring angle (red line, Eq. 5) was determined for each time-point. **B** Kymograph as in **A** but for GFP intensity of an embryo producing NMY-2::GFP. Red line corresponds to ring angle determined from kymograph as in A and defined in Eq. 5, whereas for the blue line the DV velocity from PIV was used yielding a ring angle as defined in Eq. 6

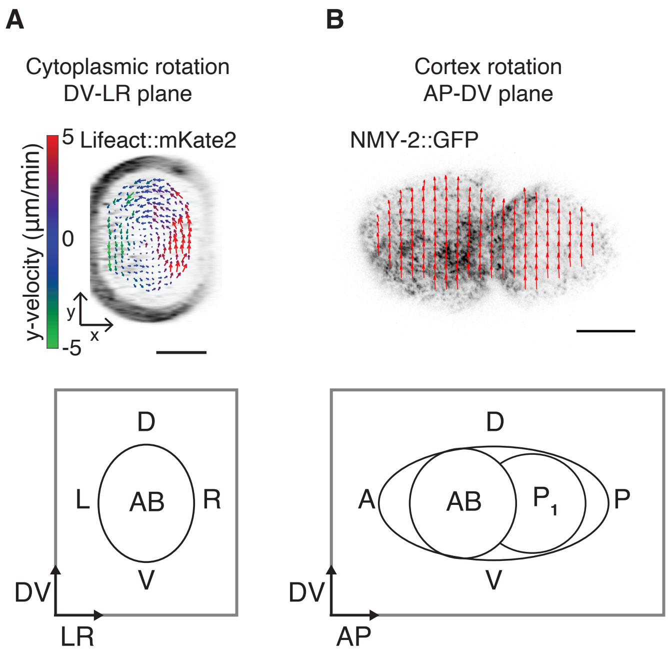

Fig. S6. Both the whole AB cell and the whole embryo rotate during AB cytokinesis.
A Still image of the AB cell of a compressed embryo producing lifeact-mKate2, in the DV-LR plane, undergoing tension-driven rotation. The mean flow field, as measured by particle image velocimetry (PIV) on cytoplasmic F-actin signal, is overlaid. Velocity vectors are color-coded for the component along the future DV axis (y-velocity). The flow field reveals that the cytoplasm rotates in the same direction as the mitotic spindle and cytokinetic ring. **B** Still image of a compressed two-cell embryo producing NMY-2::GFP, in the AP-DV plane, undergoing tension-driven rotation. The mean flow field, as measured by particle image velocimetry (PIV), on cortical NMY-2::GFP signal is overlaid. The AB cell and the neighboring P1 cell rotate in the same direction at the same time. Scale bars = 10 μm.

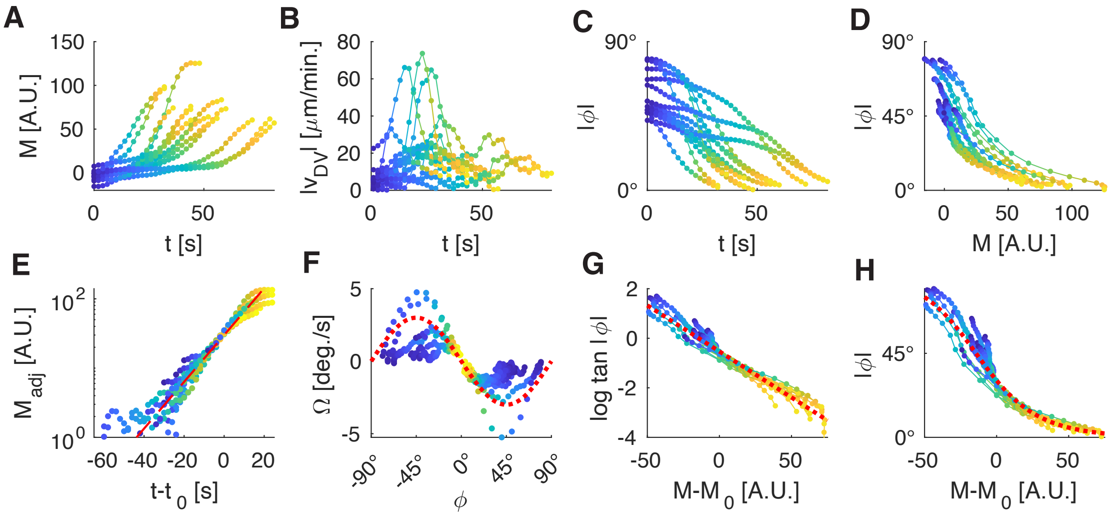

Fig. S7. Quantification of ring dynamics and comparison to theory using NMY-2-GFP All data points are time points from single embryos. Blue and yellow points correspond to first and last time point of rotation. Connected points correspond to subsequent time-points of single embryo. **A**: Trajectories of the ring intensity M_{raw} (Eq. 7). **B**: Average DV velocity as determined using PIV. **C**: Trajectories of the ring angle (Eq. 6) **D**: Ring angle plotted as a function of the ring intensity M_{raw}. **E**: Lin-log plot of the time evolution of the adjusted ring intensity M. Allowing for a constant offset (Eq. 9), we find that the ring intensities grow exponentially for early time points with a common growth rate $\lambda = 1/(13s)$. Red dashed line corresponds to exponential fit. **F**: Cortical angular velocity Ω as a function of the ring angle ϕ. We find that the sign of the angular velocity is always opposite to the sign of the angle such that $\phi = 0^\circ$ corresponds to a stable fixed point and $\phi = 90^\circ$ to an unstable fixed point of the dynamics. As expected from a coarse-grained model yielding $\Omega \sim -\sin 2\,\phi$ (Eq. 80), angular velocities are maximal close to 45°. Red dotted line is $-3\sin 2\phi$. **G**: Plot of $\log|\tan\phi\,(t)\,|$ as a function of the relative ring intensity $M_{\mathrm{raw}}(t) - M_{\mathrm{raw}}(\phi(t) = 30^\circ$ reveals that curves from different embryos collapse onto linear curve (red dotted line) with common slope as expected from the coarse-grained model for a linear relationship between Myosin concentration and active surface tension. **H**: Same as in **G**: but plotting $|\phi\,(t)|$. Red dotted curve is given by Eq. 10

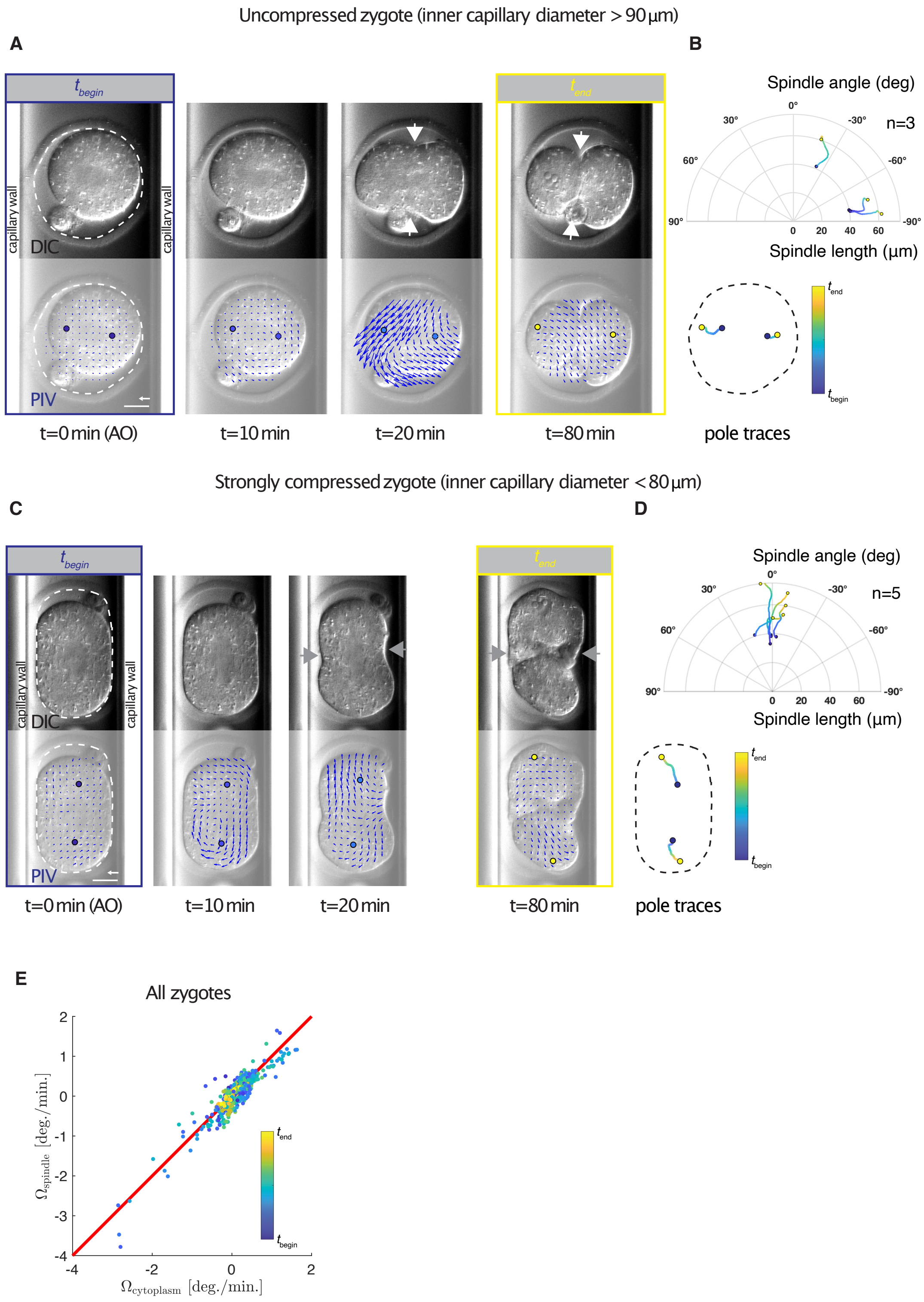

Fig. S8. Mouse zygote division upon different levels of compression Mouse zygote division inside glass capillaries in **A-B** the absence of compression (inner capillary diameter >= 90 μm) and in **C-D** the presence of strong compression (inner capillary diameter < 80 μm). Dashed line indicates the shape of the zona pellucida. Arrow heads mark the cytokinetic ring. Lower panels in **A,C** show the cytoplasmic flow field, measured by PIV, overlaid. Spindle poles, marked with filled circles, were manually identified at anaphase onset (left), and subsequently tracked automatically over time by using the interpolated local flow field (see methods). Spindle pole time traces of the displayed zygote are shown on the right. In strongly compressed zygotes **C-D** the mitotic spindle already aligns during metaphase, and stays aligned during anaphase. n=number of embryos. AO = anaphase onset. PIV vector scale bar = 1 μm/min. Scale bar = 20 $\mu\mathrm{m}$. **E**: Scatter plot of the angular velocities of the outer most layer of the cytoplasm ($\Omega_{\mathrm{cytoplasm}}$ as defined in Eq. 11) and the spindle axis ($\Omega_{\mathrm{spindle}}$ defined by the spindle poles in **B,D** and main Fig. 5B). Each point corresponds to a single time point (time resolution: 2min.) in a single embryo.

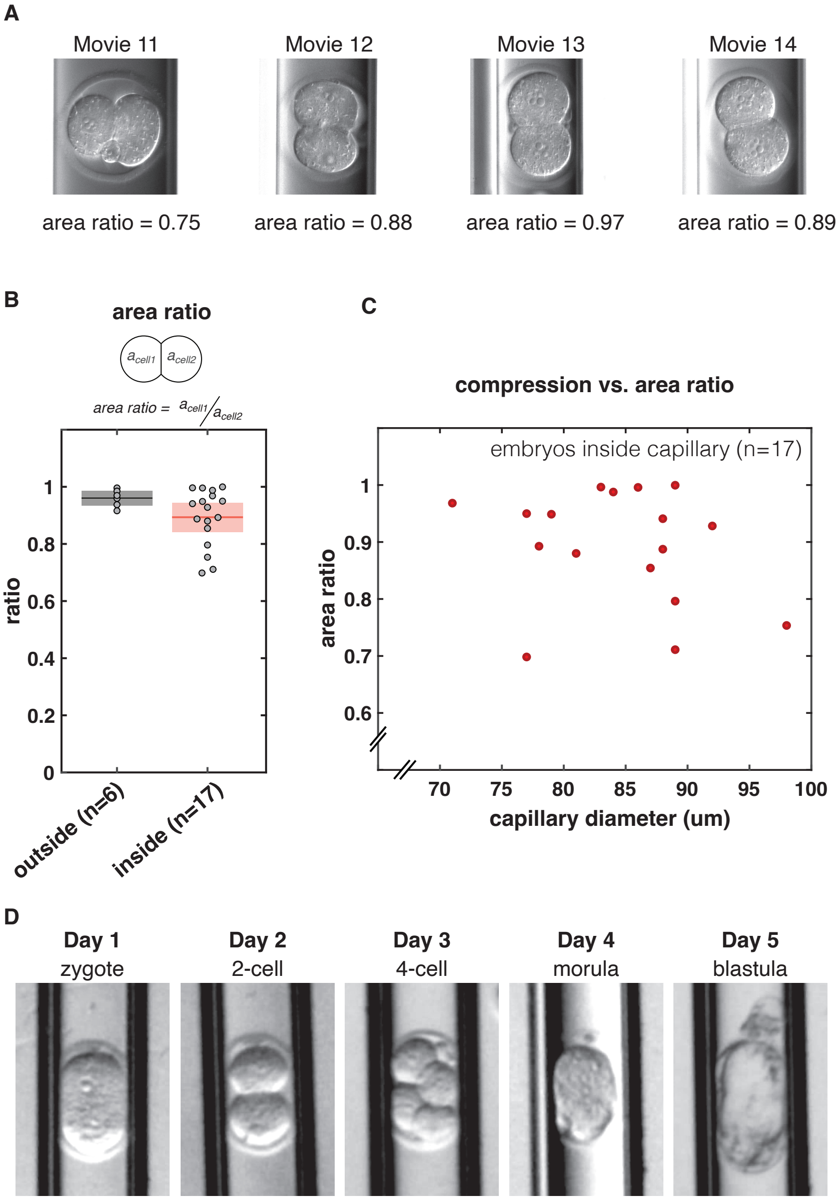

Fig. S9. Mouse zygote divisions inside a capillary can display asymmetry A Still images of the 2-cell embryos that arose from the zygote divisions shown in Movie 11-14. Some embryos that were cultured and imaged inside the glass capillary displayed an asymmetric first division. This asymmetry was quantified by measuring the area ratio, as defined in **B**. **B** Area ratio in embryos cultured outside and inside glass capillaries. The area ratio is defined as the ratio of the areas of both daughter cells. The area ratio was measured from still images of 2-cell embryos during interphase (like those shown in **A**). Data points represent individual embryos. For the embryos cultured inside capillaries, we obtained the ratios from the time lapse imaging data set displayed in Fig. 5 and Fig. S8. One embryo was excluded from this analysis because it moved out of the field of view after cytokinesis. The control embryos cultured outside the capillaries were not exposed to time lapse imaging. Although no significant difference in the median was observed for the area ratio (Wilcoxon rank sum test, pval=0.1952), the variance was significantly larger in the embryos developing inside capillaries (Bartlett test, pval=0.011). This indicates that embryos developing inside capillaries can display asymmetric cell division. **C** Area ratio plotted over capillary diameter. Data points represent individual embryos developing inside capillaries. No correlation between compression strength and cell division asymmetry was observed (Pearson correlation test, pval=0.34). **D** Embryo inside a capillary developing into a blastula over the course of 5 days. In total 8 embryos developing inside capillaries were monitored over 5 consecutive days. All of these developed into a morula, and 6 out of 8 developed into a blastocyst and hatched (right image).

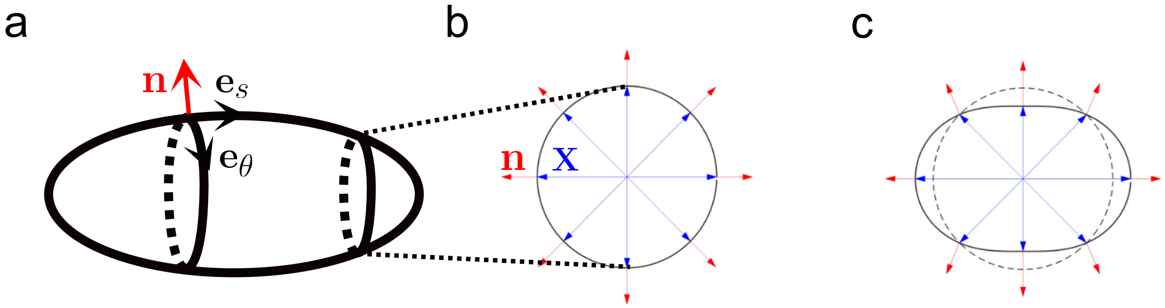

Fig. S10. Geometry of an almost axisymmetric surface. (a) axisymmetric surface with tangential vectors $\mathbf{e}_s$, $\mathbf{e}_\theta$ and normal vector $\mathbf{n}$. (b) Cross-section (xy plane) of axisymmetric surface is circle such that position vector $\mathbf{X}$ is parallel to $\mathbf{n}$ in this plane. Hence, $\mathbf{z} \cdot (\mathbf{X} \times \mathbf{n}) = 0$. (c) Upon non-axisymmetric deformation of the surface, e.g. due to compression of the embryo, the cross-section becomes non-circular and $\mathbf{z} \cdot (\mathbf{X} \times \mathbf{n}) \neq 0$ in general.

Movie S1. Dynamics of the mitotic spindle and actin cortex during AB cell division in an uncompressed embryo. Spinning disc imaging of an uncompressed embryo producing Lifeact::mKate2 and GFP::tubulin. 3D imaging was performed by making 30 μm z-stacks (dz=1um) capturing the entire embryo. Subsequently, maximum intensity projections were made to visualize the DV-LR plane (left panels) and the AP-DV plane (right panels). Due to the lower axial resolution in the z-direction, pixels along the z-axis (LR axis) were interpolated on a grid with 0.1058 μm spacing corresponding to the pixel size in the x- and y-axis (AP and DV respectively). Top panels show inverted GFP::tubulin channel alone and bottom panels show the merged Lifeact-mKate2 and GFP::tubulin channels.

Movie S2. Dynamics of the mitotic spindle and cortex during AB cell division in a compressed embryo. Spinning disc imaging of a compressed embryo producing Lifeact::mKate2 and GFP::tubulin. 3D imaging was performed by making 25 μm z-stacks (dz=1um) capturing the entire embryo. Rest is as in Movie 1.

Movie S3. Dynamics of the mitotic spindle during AB cell division in a compressed *lin-5(RNAi)* embryo viewed in the DV-LR plane. Movie shows GFP::tubulin signal in the AB cell projected onto the DV-LR plane in *lin-5(RNAi)* embryo producing GFP::tubulin and endogenously labeled LIN-5::ePDZ::mCherry (not visualized).

Movie S4. Dynamics of the mitotic spindle during AB cell division in a compressed L4440 control embryo viewed in the DV-LR plane. Movie shows GFP::tubulin signal in the AB cell projected onto the DV-LR plane in an L4440 control embryo producing GFP::tubulin and endogenously labeled LIN-5::ePDZ::mCherry (not visualized).

Movie S5. LIN-5 localization in the two-cell embryo during AB anaphase progression. Movie shows endogenously labeled LIN-5::ePDZ::mCherry in a (left) control embryo that shows initial long axis alignment, a (middle) control embryo that shows initial short axis alignment, and an (right) *opto-lin-5* embryo upon global blue-light illumination. Top row shows the embryo midplanes, bottom row shows the cortical planes. The first time frame in the *opto-lin-5* embryo (right) is captured prior to global blue-light illumination. From the second time frame onwards, LIN-5::ePDZ::mCherry displays strong cortical enrichment in the AB and P1 cell. See Fig. S3 and its caption for further details.

Movie S6. Dynamics of the mitotic spindle during AB cell division in a compressed *opto-lin-5* embryo viewed in the DV-LR plane. Movie shows GFP::tubulin signal in the AB cell projected onto the DV-LR plane in an *opto-lin-5* embryo producing GFP::tubulin, PH::GFP::LOV2 and endogenously labeled LIN-5::ePDZ::mCherry (not visualized).

Movie S7. Dynamics of the mitotic spindle during AB cell division in a compressed control embryo at 25C viewed in the DV-LR plane. Movie shows GFP::tubulin signal in the AB cell projected onto the DV-LR plane in a control embryo producing GFP::tubulin and Lifeact::mKate2 (not visualized).

Movie S8. Dynamics of the mitotic spindle during AB cell division in a compressed *nmy-2(ts)* embryo at 25C viewed in the DV-LR plane. Movie shows GFP::tubulin signal in the AB cell projected onto the DV-LR plane in an *nmy-2(ts)* embryo producing GFP::tubulin and Lifeact::mKate2 (not visualized).

Movie S9. Dynamics of the mitotic spindle during AB cell division in a compressed *opto-lin-5; nmy-2(ts)* embryo and its controls at 25C. Movie panels show GFP::tubulin signal in the AB cell projected onto the DV-LR plane in *opto-lin-5* (left), *nmy-2(ts)* (middle) and *opto-lin-5; nmy-2(ts)* (right) embryos imaged at 25C. Because expression levels of GFP::tubulin varied, the contrast in the movies was differently adjusted.

Movie S10. Cortical movements in the AB cell during whole embryo rotation viewed in the AP-DV plane. Top panel: Cortical rotation of an embryo producing endogenously labeled NMY-2::GFP, imaged using high time resolution (dt=2s). Only the cortical surface was imaged in the AP-DV plane and displayed as inverted color. Bottom panel: Same embryo with the flow field, measured using Particle Image Velocimetry (PIV), overlaid. Both the anterior AB cell and the neighboring P1 cell undergo a similar rotation within the stationary egg shell. Scale bar=10 μm.

Movie S11. Uncompressed mouse zygote undergoing division inside a glass capillary. Differential Interference Contrast (DIC) movie of a non-compressed mouse zygote undergoing cell division inside a glass capillary, focussed on the central plane of the embryo. The cytoplasmic flow field in each timepoint, as measured by PIV, is overlaid (right side). The positions of the spindle poles were manually marked in the first time point

at anaphase onset, and their approximate movement was subsequently inferred using the local cytoplasmic flow field. No reorientation of the cell division axis is observed.

Movie S12. Slightly compressed mouse zygote undergoing division inside a glass capillary. Differential Interference Contrast (DIC) movie of a slightly compressed mouse zygote undergoing cell division inside a glass capillary, focussed on the central plane of the embryo. The cytoplasmic flow field and the inferred spindle pole positions were overlaid as in Movie 11. The cell division axis reorients during anaphase in order to ensure Hertwig's rule execution.

Movie S13. Strongly compressed mouse zygote undergoing division inside a glass capillary. Differential Interference Contrast (DIC) movie of a strongly compressed mouse zygote undergoing cell division inside a glass capillary, focussed on the central plane of the embryo. The cytoplasmic flow field and the inferred spindle pole positions were overlaid as in Movie 11. The cell division axis is already aligned along the long axis prior to anaphase and remains aligned.

Movie S14. Slightly compressed mouse zygote undergoing division inside a glass capillary. Differential Interference Contrast (DIC) movie of a slightly compressed mouse zygote undergoing cell division inside a glass capillary, focussed on the central plane of the embryo. The cytoplasmic flow field and the inferred spindle pole positions were overlaid as in Movie 11. This embryo undergoes a rotation towards alignment during ring ingression, but does not reach proper long axis alignment during cytokinesis. However, after cytokinesis the two-cell embryo continues a slow rotation into long axis alignment, presumably due to space constraints. We observed such behavior in 2 out of 10 mildly compressed embryos.

References

1. J Liu, LL Maduzia, M Shirayama, CC Mello, Nmy-2 maintains cellular asymmetry and cell boundaries, and promotes a src-dependent asymmetric cell division. *Dev. biology* **339**, 366–373 (2010).
2. DJ Dickinson, JD Ward, DJ Reiner, B Goldstein, Engineering the caenorhabditis elegans genome using cas9-triggered homologous recombination. *Nat. methods* **10**, 1028–1034 (2013).
3. LE Fielmich, et al., Optogenetic dissection of mitotic spindle positioning in vivo. *Elife* **7**, e38198 (2018).
4. AC Reymann, F Staniscia, A Erzberger, G Salbreux, SW Grill, Cortical flow aligns actin filaments to form a furrow. *Elife* **5**, e17807 (2016).
5. E Stamhuis, W Thielicke, Pivlab–towards user-friendly, affordable and accurate digital particle image velocimetry in matlab. *J. open research software* **2**, 30 (2014).
6. G Salbreux, F Jülicher, Mechanics of active surfaces. *Phys. Rev. E* **96**, 032404 (2017).
7. A Mietke, V Jemseena, KV Kumar, IF Sbalzarini, F Jülicher, Minimal model of cellular symmetry breaking. *Phys. review letters* **123**, 188101 (2019).
8. LG Pimpale, TC Middelkoop, A Mietke, SW Grill, Cell lineage-dependent chiral actomyosin flows drive cellular rearrangements in early *Caenorhabditis elegans* development. *eLife* **9**, e54930 (2020).
